# Androgen receptor-regulated ZNF419 modulates ferroptosis sensitivity via the ZDHHC20–GPX4 pathway in prostate cancer

**DOI:** 10.1016/j.redox.2026.104433

**Published:** 2026-10-08

**Authors:** Yuanxiang Hou, Wenyan Zhao, Jingyi Liu, Ruiping Tang, Tong Deng, Huaiyu Zhu, Hiroshi Miyamoto, Yuyan Zhu, Ming Sun, Guiyang Jiang

**Affiliations:** a Department of Urology, The Second Affiliated Hospital of Hainan Medical University, Haikou, Hainan, China; b Department of General Surgery, Shengjing Hospital of China Medical University, Shenyang, Liaoning, China; c China Medical University, Shenyang, Liaoning, China; d Department of Pathology, The College of Basic Medical Sciences and the First Hospital of China Medical University, Shenyang, Liaoning, China; e Departments of Pathology & Laboratory Medicine and Urology, University of Rochester Medical Center, Rochester, NY, USA; f Department of Urology, The First Hospital of China Medical University, Shenyang, 110001, China; g Department of Urology, Shengjing Hospital of China Medical University, Shenyang, Liaoning, China

**Keywords:** Prostate cancer, Androgen receptor, ZNF419, Ferroptosis, ZDHHC20, GPX4, Enzalutamide, AR-targeted therapy

## Abstract

Ferroptosis, an iron-dependent form of regulated cell death driven by lipid peroxidation, has recently been recognized as a crucial determinant of therapeutic response in prostate cancer (PCa). However, how androgen receptor (AR) signaling regulates ferroptotic susceptibility remains poorly understood. Here, we identify ZNF419, a previously uncharacterized zinc finger protein, as an AR-regulated factor associated with reduced ferroptosis susceptibility in PCa cells. Transcriptomic profiling and functional validation revealed that *ZNF419* expression is markedly elevated in PCa and correlates with poor clinical outcome. Promoter-proximal AR occupancy was detected at predicted ARE-containing regions of the ZNF419 locus and contributed to ZNF419 promoter activation in reporter assays, whereas enzalutamide treatment or AR knockdown reduced ZNF419 expression and increased ferroptosis-associated vulnerability. Mechanistically, ZNF419 promotes *ZDHHC20* expression and is associated with activation of a candidate downstream enhancer region. ZDHHC20, a palmitoyltransferase, contributes to GPX4 palmitoylation-associated stabilization and limits ubiquitin-mediated proteasomal degradation of GPX4. Pharmacological interference with palmitoylation attenuates ZNF419-associated GPX4 stabilization, whereas ZDHHC20 restoration partially reverses the increased ferroptosis-associated vulnerability of ZNF419-deficient cells. Collectively, our findings support a previously unrecognized AR-associated ZNF419–ZDHHC20–GPX4 regulatory axis that links AR activity to ferroptosis-associated vulnerability. These findings provide a rationale for further investigating whether modulation of this pathway may influence responses to AR-targeted therapy in advanced PCa.

## Highlights

• AR-regulated ZNF419 links androgen signaling to ferroptosis vulnerability.
• ZNF419 promotes ZDHHC20 expression through a candidate downstream enhancer.
• ZNF419–ZDHHC20 signaling supports palmitoylation-associated GPX4 stabilization.
• ZNF419 depletion sensitizes prostate cancer cells to ferroptosis-inducing stress.
• IKE xenografts support increased tumor vulnerability after ZNF419 depletion.

## Graphical abstract

Proposed model of the AR–ZNF419–ZDHHC20–GPX4 axis in prostate cancer ferroptosis regulation.DHT-activated AR signaling increases ZNF419 expression, with promoter-proximal AR binding contributing to ZNF419 promoter activation in our experimental system. ZNF419 promotes ZDHHC20 expression through a candidate downstream enhancer region. In the proposed model, ZDHHC20-associated GPX4 palmitoylation contributes to GPX4 stabilization by limiting ubiquitin-mediated proteasomal degradation. Stabilized GPX4 is associated with reduced ferroptosis-associated oxidative stress and supports prostate cancer cell survival. Enzalutamide disrupts this pathway by inhibiting AR signaling, leading to reduced ZNF419/ZDHHC20 expression, GPX4 destabilization, and increased vulnerability to ferroptosis-inducing stress.

**Figure:**
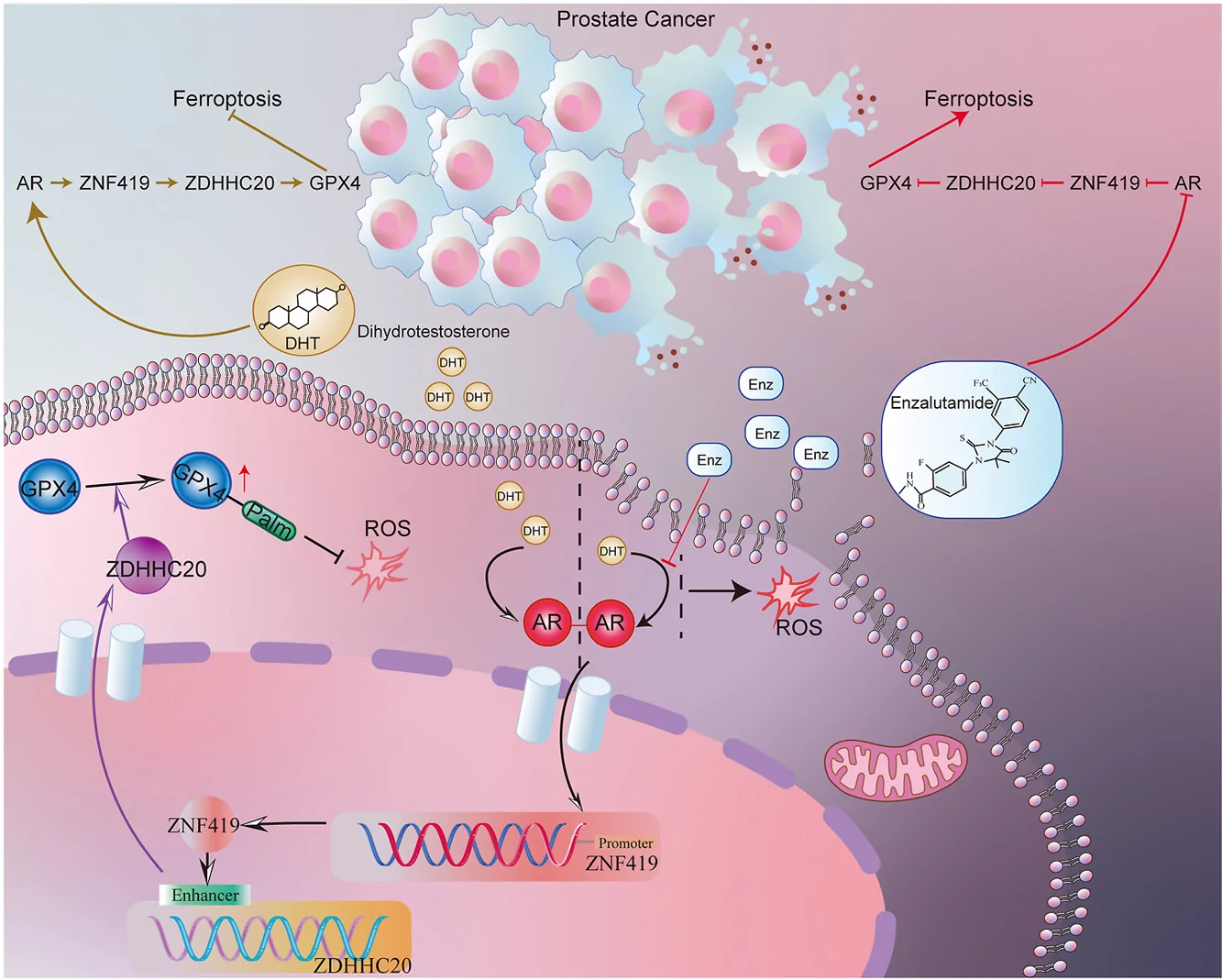

## 1 Introduction

Prostate cancer (PCa) is the most frequently diagnosed malignancy of the male genitourinary system and ranks as the leading cancer type among men in the United States, accounting for approximately 30% of all new cases annually [1]. Despite substantial advances in early detection and treatment, PCa incidence continues to rise, and more than 35,000 men die from the disease each year, posing a major public health challenge [1]. The biological behavior of PCa is remarkably heterogeneous, yet its initiation and progression are fundamentally driven by androgen receptor (AR) signaling [2,3]. Androgen deprivation therapy (ADT), achieved through surgical or pharmacologic castration, remains the cornerstone treatment for locally advanced and metastatic disease [4]. Although ADT initially induces tumor regression by suppressing androgen-dependent growth, most patients ultimately relapse with castration-resistant PCa (CRPC), a lethal stage characterized by AR reactivation and a 5-year survival rate of only 25–33% [[5], [6], [7], [8]].

The emergence of CRPC reflects diverse molecular adaptations, including *AR* amplification [9], *AR* mutations [10,11], the generation of constitutively active splice variants [12,13], and activation of bypass signaling pathways [14]. Second-generation AR antagonists such as enzalutamide have substantially improved patient survival by more effectively inhibiting AR signaling; however, resistance to these agents remains inevitable [[15], [16], [17]]. Elucidating the molecular determinants that underlie therapeutic response and resistance is therefore critical for improving treatment outcomes.

Recent studies have highlighted ferroptosis, an iron-dependent and lipid peroxidation-driven form of regulated cell death, as a crucial determinant of tumor response to therapy [18]. Increasing evidence indicates that resistance to AR-targeted agents such as enzalutamide is closely associated with suppression of ferroptosis. Preclinical studies have demonstrated that restoring ferroptotic sensitivity markedly enhances enzalutamide efficacy [[19], [20], [21], [22]]. For instance, inhibition of SLC7A11 by erastin [20], blockade of PHGDH by NCT-503 [19], or direct inhibition of GPX4 by RSL3 [22] synergizes with enzalutamide to produce pronounced antitumor effects in multiple experimental models. These findings suggest that ferroptosis represents a promising therapeutic vulnerability in PCa. However, current understanding of how enzalutamide modulates ferroptotic signaling and how AR activity regulates ferroptosis remains limited. Recent work by Sun et al. revealed that in PCa cell lines, including LNCaP, C4-2, and 22Rv1, both full-length AR (AR-FL) and *AR* splice variants (AR-Vs) occupy promoter and enhancer regions of *SLC7A11*, thereby upregulating *SLC7A11* transcription, enhancing system Xc− activity, and suppressing ferroptosis [23]. In contrast, Tang et al. reported that enzalutamide-mediated AR inhibition downregulates the ubiquitin E3 ligase NEDD4L, decreases SLC7A11 ubiquitination and degradation, and consequently reduces ferroptosis, thereby promoting therapeutic resistance in C4-2 and 22Rv1 cells [20]. These seemingly divergent findings underscore the complexity of AR-dependent ferroptosis regulation and highlight the need for further investigation. To address this issue, we sought to define AR-regulated molecular networks that determine ferroptotic sensitivity in PCa cells.

By integrating transcriptomic datasets derived from AR perturbation models, ferroptosis induction profiles, and enzalutamide-resistance signatures, we identified “*ZNF419*”, a zinc finger protein-encoding gene, as a potential link between AR signaling and ferroptosis regulation. Analysis using the ferroptosis database “FerrDb V2” [https://www.zhounan.org/ferrdb/] revealed that *ZNF419* expression increased more than twofold following erastin-induced ferroptosis, consistent with prior bioinformatic evidence implicating ZNF419 in ferroptotic processes. However, the biological function of ZNF419 in the context of pathologic conditions, particularly in PCa, has not been elucidated. These observations prompted us to further explore the potential role of ZNF419 in ferroptosis regulation.

In this study, we provide evidence that AR signaling positively regulates ZNF419 expression and that promoter-proximal AR binding contributes to ZNF419 transcriptional activation in prostate cancer cells. ZNF419, in turn, modulates ferroptosis susceptibility through a ZDHHC20-associated regulatory mechanism that contributes to GPX4 palmitoylation and stabilization, thereby supporting tumor cell survival under ferroptosis-inducing stress. Conversely, enzalutamide antagonizes AR signaling, leading to *ZNF419* downregulation, reduced GPX4 stability, enhanced ferroptosis-associated oxidative stress and increased susceptibility to ferroptosis-inducing treatments, which may contribute to enzalutamide-mediated antitumor activity. While this work does not fully define the mechanisms underlying enzalutamide resistance, delineating the AR–ZNF419–ZDHHC20–GPX4 pathway clarifies how AR activity modulates ferroptosis. These findings provide a mechanistic basis for further investigating how ferroptosis-associated vulnerability may influence responses to AR-targeted therapy.

## 2 Results

### 2.1 ZNF419 is highly expressed in PCa and its depletion enhances enzalutamide sensitivity

To elucidate how AR signaling regulates ferroptotic sensitivity and to identify novel ferroptosis-related therapeutic targets in PCa, we analyzed three public GEO datasets, including GSE248895 [24] (AR perturbation), GSE232034 [25] (erastin-treated prostate cancer cells), and GSE164880 [26] (enzalutamide-resistant versus parental cell lines). Differential-expression analysis was performed using GEO2R, and genes with a nominal P value < 0.05 and |log2 fold change| > 0.4 were retained. Complementary four-set Venn and UpSet analyses identified six genes shared across all four filtered gene sets: DDIT3, SESN2, HERPUD1, ATF3, TSC22D3, and ZNF419 (Fig. 1A and B). This intersection was treated as an exploratory candidate pool rather than as a statistical ranking. After defining the shared set, ZNF419 was prioritized through a qualitative, hypothesis-driven assessment that considered its erastin-associated regulation recorded in FerrDb V2, its prostate cancer expression and survival associations in follow-up public-dataset analyses, its plausible transcriptional regulatory potential as a zinc-finger protein, and its relatively unexplored role in PCa ferroptosis, whereas several other candidates have established stress-response or ferroptosis-related functions [[27], [28], [29], [30], [31]]. ZNF419 was not selected as the candidate with the largest effect size or smallest P value, and the other overlapping genes were not considered functionally excluded. We therefore selected ZNF419 for further functional and mechanistic analysis.

**Fig. 1:**
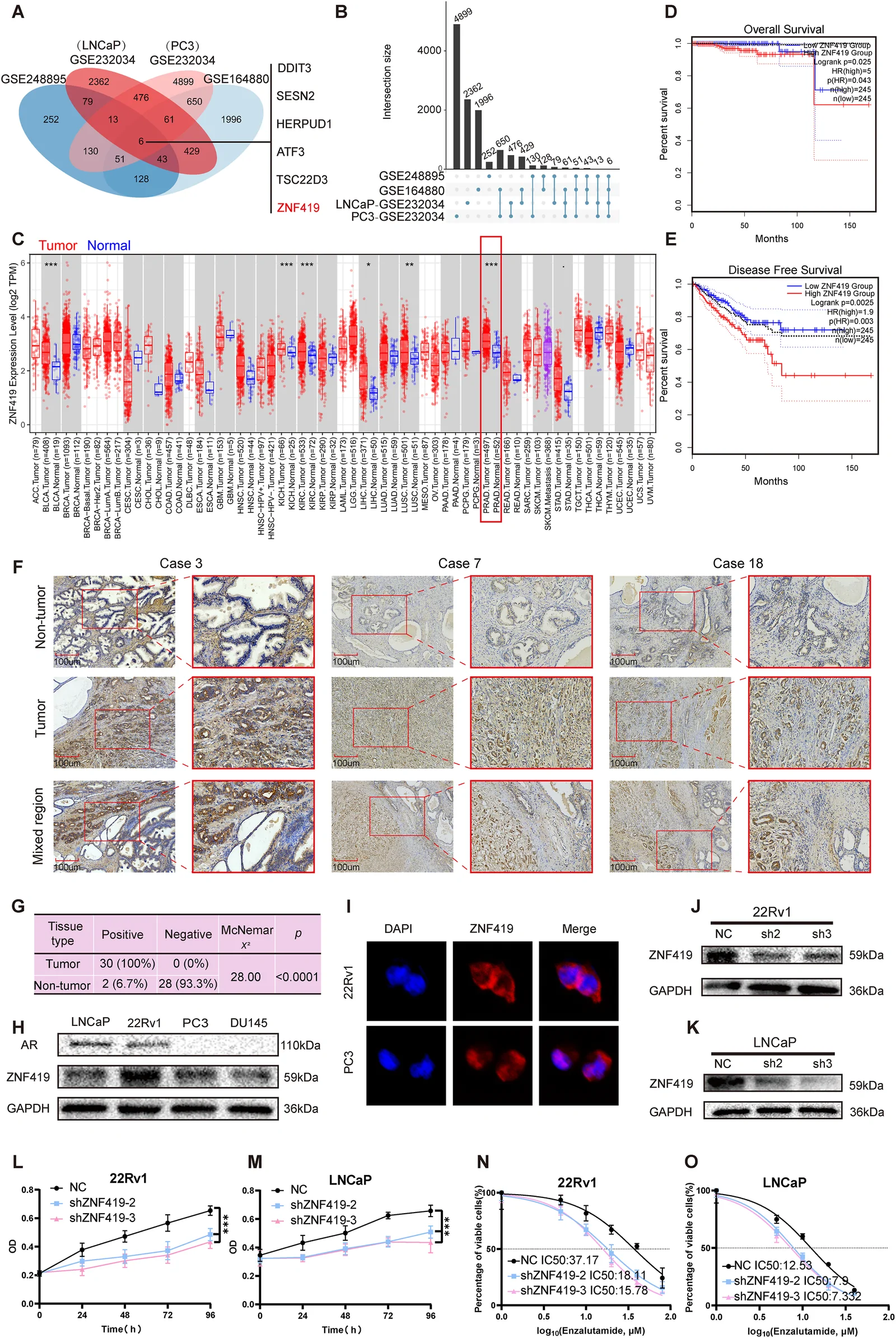
ZNF419 is highly expressed in prostate cancer and modulates enzalutamide sensitivity. (A) Four-set Venn diagram showing the overlap among the filtered gene lists derived from GSE248895, GSE164880, and the LNCaP and PC3 comparisons within GSE232034. (B) UpSet plot showing the sizes and intersection structure of the four filtered gene sets. Six genes were shared by all four gene sets: DDIT3, SESN2, HERPUD1, ATF3, TSC22D3, and ZNF419. (C) Expression of *ZNF419* across tumor types from TIMER2.0 database. (D, E) Kaplan–Meier survival analysis showing that high *ZNF419* expression correlates with poor overall and disease-free survival in prostate cancer. (F, G) Representative immunohistochemical staining of ZNF419 in adjacent non-tumor and prostate cancer tissues. (H) Western blot analysis of AR and ZNF419 expression in prostate cancer cell lines. (I) Immunofluorescence showing ZNF419 localization in 22Rv1 and PC3 cells. (J, K) Validation of ZNF419 knockdown efficiency by Western blotting in 22Rv1 and LNCaP cells. (L, M) Cell proliferation curves of control and ZNF419-depleted cells. (N, O) Dose–response curves of NC, shZNF419-2, and shZNF419-3 22Rv1 (N) and LNCaP (O) cells treated with enzalutamide at 0, 5, 10, 20, 40, or 80 μM for 48 h. IC50 values were calculated from fitted nonlinear regression curves. NC indicates the negative control shRNA group. Data are presented as mean ± SD from three independent biological experiments unless otherwise indicated. Kaplan–Meier survival curves (D, E) were compared using the log-rank test; the distribution of positive and negative ZNF419 staining in paired tumor and adjacent non-tumor specimens (G) was compared using McNemar's test (n = 30 pairs; χ^2^ = 28.00, p < 0.0001); and cell proliferation curves (L, M) using two-way ANOVA followed by Tukey's multiple-comparisons test. Other two-group and multi-group comparisons were analyzed using a two-tailed Student's t-test and one-way ANOVA followed by Tukey's post hoc test, respectively. *p < 0.05, **p < 0.01, ***p < 0.001, ****p < 0.0001; ns, not significant.

To delineate the expression profile of *ZNF419*, we interrogated the UALCAN and TIMER2.0 databases, which showed that *ZNF419* was upregulated in PCa as well as in multiple other tumor types (Fig. 1C and Supplementary Fig. 1A and B). Analysis of the GEPIA2 dataset further revealed that elevated *ZNF419* expression was associated with shorter overall survival (P = 0.025; Fig. 1D) and disease-free survival (P = 0.0025; Fig. 1E) in patients with PCa, suggesting a potential oncogenic role for ZNF419.

Immunohistochemical analysis of surgical specimens revealed increased ZNF419 staining in PCa tissues (Fig. 1F). All 30 PCa specimens exhibited positive ZNF419 staining, with the vast majority showing moderate-to-strong intensity, whereas only two adjacent non-tumor tissues showed weakly positive acinar staining (Fig. 1G; McNemar's test, *p* < 0.001). These findings indicate that ZNF419 is elevated in malignant prostatic epithelium compared with adjacent non-tumor counterparts, consistent with the bioinformatic predictions. Western blotting confirmed ZNF419 expression in both AR-positive and AR-negative PCa cell lines (Fig. 1H and Supplementary Fig. 1C), while immunofluorescence staining indicated its presence in both the cytoplasm and nucleus of 22Rv1 and PC3 cells (Fig. 1I).

Stable knockdown of ZNF419 was established in AR-positive 22Rv1 and LNCaP cells, and efficient silencing was confirmed by Western blotting (Fig. 1J, K and Supplementary Fig. 1D–F). Functional assays revealed that ZNF419 depletion significantly suppressed cell proliferation (Fig. 1L and M) and migration (Supplementary Fig. 1G) in both cell lines. Moreover, silencing ZNF419 enhanced the sensitivity of PCa cells to enzalutamide, as evidenced by a reduction in IC50 values (Fig. 1N and O).

Together, these findings suggest that ZNF419 is highly expressed in PCa and may contribute to tumor growth and enzalutamide response, supporting its selection for further mechanistic investigation.

### 2.2 Promoter-proximal AR binding contributes to transcriptional activation of *ZNF419*

Androgens are key drivers of PCa initiation and progression [32]. During our intersection analysis of multiple datasets, we observed a potential association between *ZNF419* and *AR* expression. GEPIA2 correlation analysis confirmed a significant positive relationship between *AR* and *ZNF419* transcript levels (Fig. 2A).

**Fig. 2:**
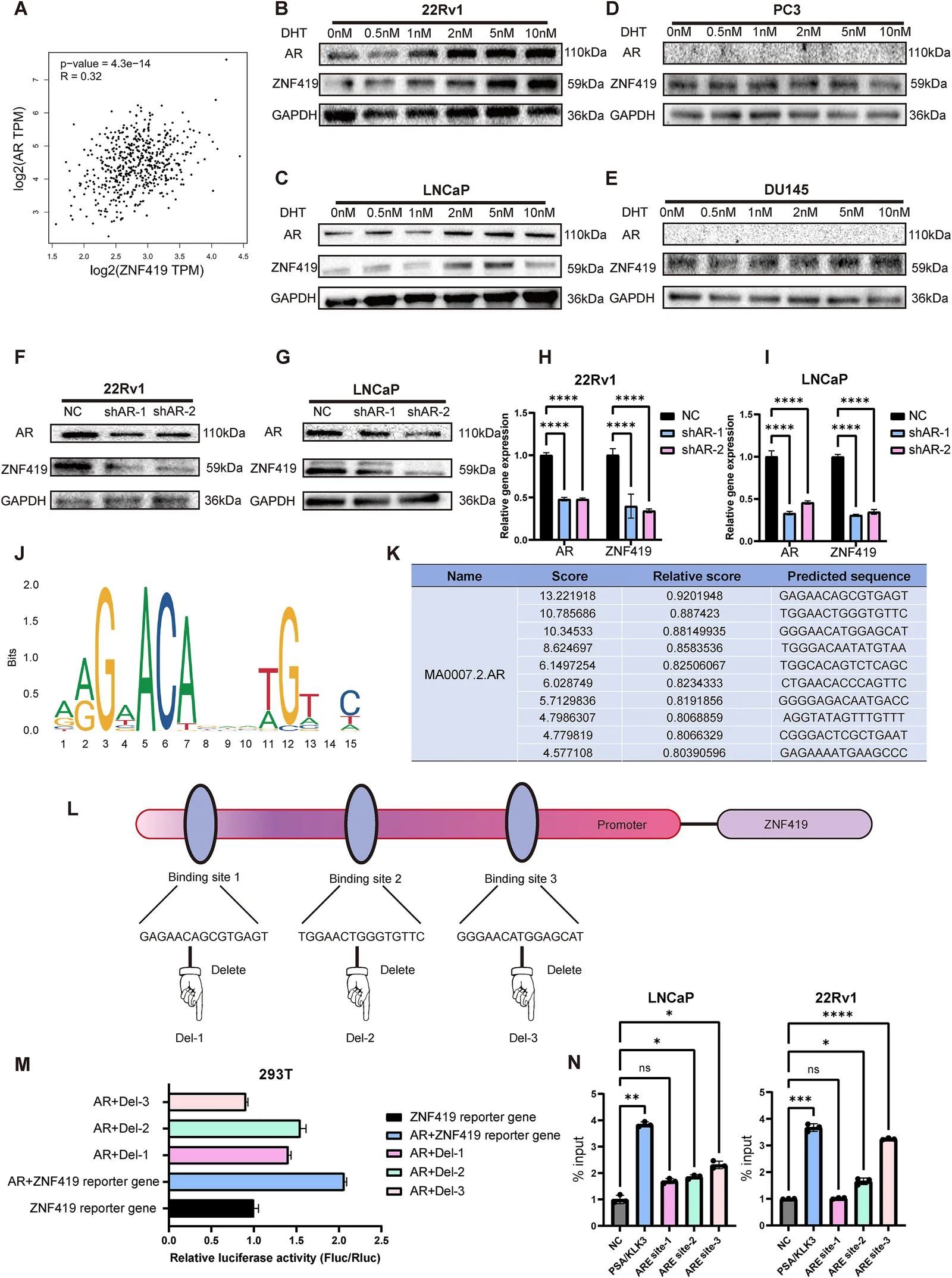
Promoter-proximal AR occupancy contributes to AR-associated transcriptional activation of ZNF419 in prostate cancer cells. (A) Correlation analysis between *ZNF419* and *AR* transcript levels in prostate adenocarcinoma from the GEPIA2 database. (B, C) Western blot analysis of AR and ZNF419 protein levels in AR-positive 22Rv1 and LNCaP cells treated with DHT at 0, 0.5, 1, 2, 5, or 10 nM for 24 h. (D, E) Western blot analysis of AR and ZNF419 protein levels in AR-negative PC3 and DU145 cells treated with DHT at 0, 0.5, 1, 2, 5, or 10 nM for 24 h. (F, G) Western blot analysis showing that AR knockdown reduced ZNF419 protein levels in 22Rv1 and LNCaP cells. (H, I) RT-qPCR analysis showing reduced *ZNF4*19 mRNA expression after *AR* knockdown in 22Rv1 and LNCaP cells. (J, K) Prediction of putative androgen response elements (AREs) within the *ZNF419* promoter using the JASPAR database. Panel J shows the AR-binding motif sequence logo, and panel K shows top-ranked predicted binding sites with corresponding scores and sequences. (L) Schematic illustration of the *ZNF419* promoter region containing three predicted ARE sites and corresponding deletion constructs (Del-1, Del-2, and Del-3) used for luciferase reporter assays. (M) Dual-luciferase reporter assays performed in 293T cells with ectopic AR expression to assess AR responsiveness of the cloned *ZNF419* promoter constructs in a controlled reporter system. Deletion of individual AREs attenuated AR-mediated promoter activation, with Del-3 showing the most pronounced reduction among the tested promoter elements. (N) AR ChIP-qPCR analysis showing AR enrichment at the predicted ARE-containing regions of the *ZNF419* promoter in LNCaP and 22Rv1 cells. The *PSA*/*KLK3* locus was included as a positive-control region. Data are presented as mean ± SD from three independent biological experiments unless otherwise indicated. Correlation analysis in panel A was performed using Pearson's correlation test. RT-qPCR data in panels H and I were analyzed using two-way ANOVA followed by Tukey's multiple-comparisons test. Luciferase reporter data in panel M were analyzed using one-way ANOVA followed by Tukey's multiple-comparisons test. ChIP-qPCR data in panel N were analyzed using repeated-measures one-way ANOVA with Geisser–Greenhouse correction followed by Dunnett's multiple-comparisons test. *p < 0.05, **p < 0.01, ***p < 0.001, ****p < 0.0001; ns, not significant.

To examine whether ZNF419 is regulated by AR signaling, AR-positive PCa cell lines, including 22Rv1 and LNCaP, were treated with increasing concentrations of dihydrotestosterone (DHT). Western blot analysis revealed that ZNF419 protein expression was markedly upregulated upon DHT stimulation, peaking at 5 nM (Fig. 2B, C and Supplementary Fig. 2A and B). In contrast, DHT treatment did not markedly alter ZNF419 levels in AR-negative PC3 and DU145 cells (Fig. 2D, E and Supplementary Fig. 2C and D).

Silencing AR in 22Rv1 and LNCaP cells significantly reduced ZNF419 protein (Fig. 2F, G and Supplementary Fig. 2E and F) and mRNA (Fig. 2H and I) expression, suggesting that AR signaling positively regulates ZNF419 expression at the transcript level. To further elucidate the mechanism by which AR regulates ZNF419 transcription, we analyzed publicly available AR ChIP-seq data from GEO sample accession GSM980647 [33] across the genomic region surrounding the *ZNF419* locus. AR occupancy signals were observed across the broader ZNF419 genomic neighborhood, including intergenic and neighboring-gene regions. Within 100 kb upstream and downstream of the ZNF419 TSS, a promoter-proximal AR enrichment signal was detected near the ZNF419 promoter region (Supplementary Fig. 2G). Additional distal AR peaks were also observed, suggesting that distal AR-bound regulatory elements may potentially contribute to ZNF419 regulation.

Because the promoter-proximal region overlapped the *ZNF419* promoter/TSS and could be directly evaluated using promoter reporter assays, ARE-deletion analysis, and ChIP-qPCR, we focused our functional validation on this region and performed motif prediction using the JASPAR database. Three putative androgen response elements (AREs) with high predicted binding scores were identified within the proximal promoter of *ZNF419* (Fig. 2J and K). To functionally evaluate these predicted elements, we generated luciferase reporter constructs containing either the wild-type *ZNF419* promoter or mutant variants with deletions of each predicted ARE (Del-1, Del-2, and Del-3; Fig. 2L). To assess whether the *ZNF419* promoter is responsive to AR in a controlled reporter system, we performed dual-luciferase reporter assays in 293T cells with ectopic AR expression. AR co-expression significantly enhanced ZNF419 promoter activity, whereas deletion of individual AREs attenuated this effect, with Del-3 showing the most pronounced reduction among the tested promoter elements (Fig. 2M).

Consistent with these reporter data, ChIP-qPCR confirmed AR enrichment at the predicted ARE-containing regions of the ZNF419 promoter in LNCaP and 22Rv1 cells, with the strongest enrichment observed at site 3 (Fig. 2N). The canonical AR target locus PSA/KLK3 was included as a positive-control region. Together, these results support that promoter-proximal AR occupancy functionally contributes to AR-associated transcriptional activation of ZNF419 in our experimental system. Among the tested promoter elements, the third predicted ARE site (GGGAACATGGAGCAT) showed the strongest AR enrichment and the most pronounced effect on promoter activity.

### 2.3 ZNF419 knockdown enhances ferroptosis sensitivity in PCa cells

To explore the functional role of ZNF419 in PCa progression and treatment response, we examined its impact on ferroptosis sensitivity. In both 22Rv1 and LNCaP cells, ZNF419 knockdown reduced cell viability following enzalutamide treatment, and this effect was effectively rescued by the ferroptosis inhibitor ferrostatin-1 (Fer-1) (Fig. 3A and B). In contrast, neither the apoptosis inhibitor Z-VAD-FMK nor the necroptosis inhibitor necrostatin-1 (Nec-1) produced comparable rescue, suggesting a substantial Fer-1-sensitive component of the observed cytotoxic response.

**Fig. 3:**
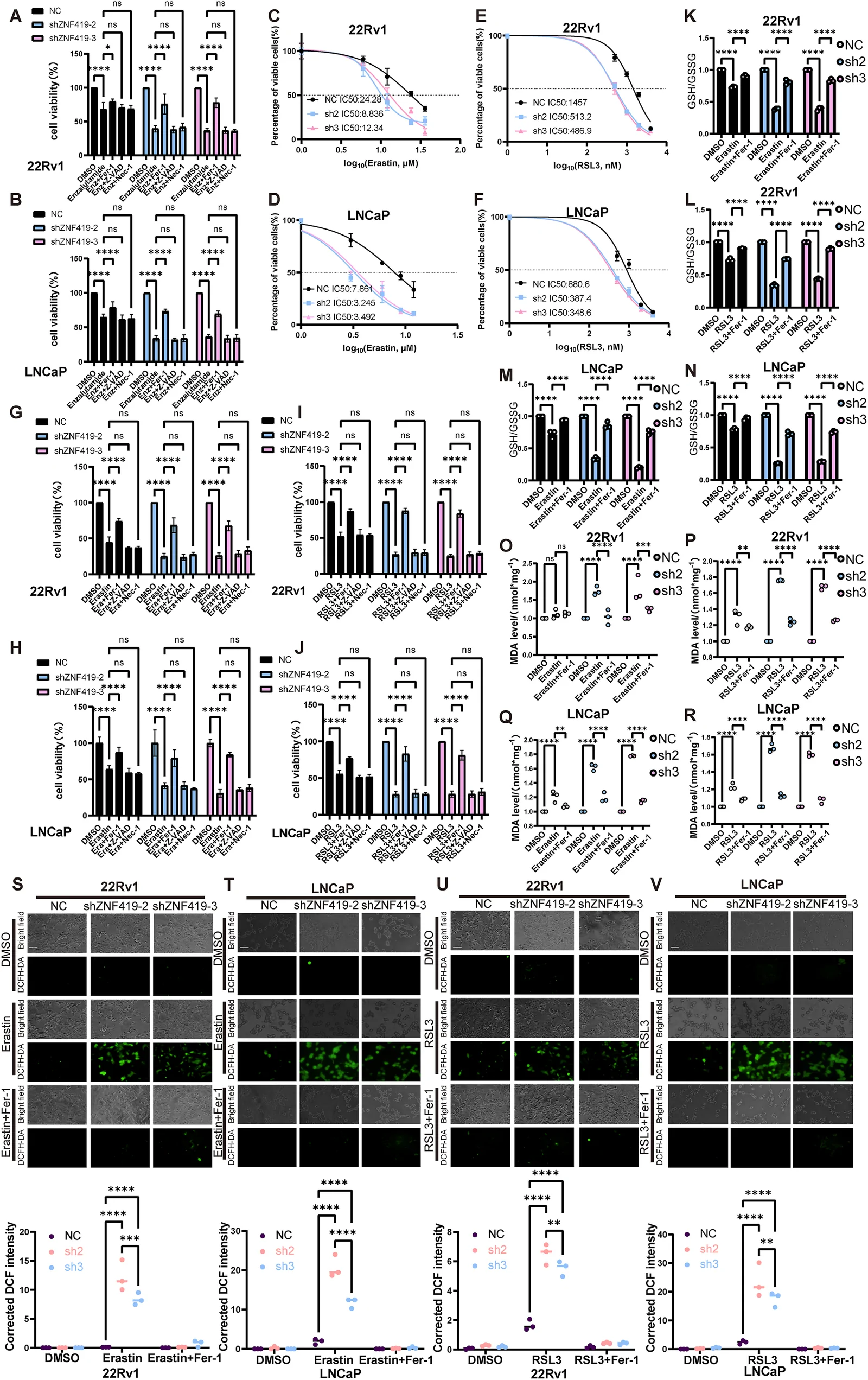
ZNF419 depletion enhances ferroptosis-associated cytotoxicity and sensitizes prostate cancer cells to enzalutamide and ferroptosis inducers. (A, B) Cell viability of NC, shZNF419-2, and shZNF419-3 22Rv1 (A) and LNCaP (B) cells treated with ENZ (10 μM) alone or co-treated with Fer-1 (10 μM), Z-VAD-FMK (10 μM), or Nec-1 (10 μM) for 48 h. (C, D) Dose–response curves of NC, shZNF419-2, and shZNF419-3 22Rv1 (C) and LNCaP (D) cells treated with erastin at 0, 6, 12, 24, or 36 μM for 24 h. (E, F) Dose–response curves of NC, shZNF419-2, and shZNF419-3 22Rv1 (E) and LNCaP (F) cells treated with RSL3 at 0, 0.5, 1, 2, or 4 μM for 24 h. IC50 values were calculated from fitted nonlinear regression curves. (G, H) Cell viability of NC, shZNF419-2, and shZNF419-3 22Rv1 (G) and LNCaP (H) cells treated with erastin (10 μM) alone or co-treated with Fer-1 (10 μM), Z-VAD-FMK (10 μM), or Nec-1 (10 μM) for 24 h. (I, J) Cell viability of NC, shZNF419-2, and shZNF419-3 22Rv1 (I) and LNCaP (J) cells treated with RSL3 (1 μM) alone or co-treated with Fer-1 (10 μM), Z-VAD-FMK (10 μM), or Nec-1 (10 μM) for 24 h. (K–N) Intracellular GSH/GSSG ratios in control and ZNF419-depleted cells following treatment with erastin or RSL3, with or without Fer-1 co-treatment. Panels K and L show 22Rv1 cells, whereas panels M and N show LNCaP cells. (O–R) Malondialdehyde (MDA) levels in control and ZNF419-depleted cells following treatment with erastin or RSL3, with or without Fer-1 co-treatment. Panels O and P show 22Rv1 cells, whereas panels Q and R show LNCaP cells. Individual data points represent independent biological experiments. (S, T) Representative fluorescence microscopy images of DCFH-DA staining and corresponding quantification of corrected DCF fluorescence intensity in 22Rv1 (S) and LNCaP (T) cells following erastin treatment, with or without Fer-1 rescue. (U, V) Representative fluorescence microscopy images of DCFH-DA staining and corresponding quantification of corrected DCF fluorescence intensity in 22Rv1 (U) and LNCaP (V) cells following RSL3 treatment, with or without Fer-1 rescue. Data are presented as mean ± SD from three independent biological experiments. Cell-viability data in panels A, B, and G–J, redox-related biochemical data in panels K–R, and ROS fluorescence quantification in panels S–V were analyzed using two-way ANOVA followed by Tukey's multiple-comparisons test. Dose–response curves in panels C–F were fitted using nonlinear regression to estimate IC50 values, and group differences across the dose–response datasets were analyzed using two-way ANOVA followed by Tukey's multiple-comparisons test. Panels S–V include representative fluorescence images and corresponding quantitative data from three independent biological experiments. *p < 0.05, **p < 0.01, ***p < 0.001, ****p < 0.0001; ns, not significant.

Consistently, ZNF419-depleted cells exhibited enhanced sensitivity to ferroptosis inducers. The half-maximal inhibitory concentrations (IC50) of erastin (Fig. 3C and D) and RSL3 (Fig. 3E and F) were decreased in both 22Rv1 and LNCaP cells upon ZNF419 silencing. Treatment with Fer-1, but not Z-VAD-FMK or Nec-1, significantly rescued the reduction in cell viability induced by erastin (Fig. 3G and H) or RSL3 (Fig. 3I and J). To determine whether ZNF419 depletion alters RSL3 sensitivity beyond a single-dose rescue setting, we performed additional 24-h RSL3 dose–response assays in the absence or presence of Fer-1. In the absence of Fer-1, ZNF419 knockdown shifted the RSL3 dose–response curves leftward. The estimated IC50 values in 22Rv1 cells were 1.66 μM for NC, 0.40 μM for shZNF419-2, and 0.50 μM for shZNF419-3; the corresponding values in LNCaP cells were 1.05, 0.41, and 0.37 μM, respectively. Fer-1 restored cell viability across multiple RSL3 concentrations and shifted the curves rightward. Because 50% viability was not reached in the Fer-1-containing groups within the tested RSL3 range of 0–4 μM, their fitted IC50 estimates were considered model-based extrapolations rather than precise experimentally bounded values (Supplementary Fig. 3A and B). Together with the supportive redox changes described below, these results indicate that ZNF419 depletion increases susceptibility to treatments that induce ferroptosis-associated stress, with a substantial Fer-1-sensitive component.

Consistent with these functional results, ZNF419 knockdown decreased the GSH/GSSG ratio (Fig. 3K–N) and increased MDA levels (Fig. 3O–R) under ferroptosis-inducing conditions. DCFH-DA fluorescence imaging further showed increased general ROS accumulation in ZNF419-deficient 22Rv1 and LNCaP cells following erastin or RSL3 treatment, whereas Fer-1 attenuated these changes. Quantification of corrected DCF fluorescence intensity confirmed the increased ROS signals observed in ZNF419-depleted cells (Fig. 3S–V). Together, these biochemical and fluorescence-based findings further support enhanced ferroptosis-associated oxidative stress following ZNF419 depletion.

Collectively, these findings indicate that ZNF419 depletion increases the susceptibility of PCa cells to enzalutamide and ferroptosis inducers, supporting a role for ZNF419 in reducing susceptibility to ferroptosis-inducing treatments in PCa cells.

### 2.4 ZNF419 regulates ferroptosis sensitivity in PCa cells via GPX4 stabilization

To further elucidate the molecular mechanism by which ZNF419 modulates ferroptosis sensitivity, we examined its impact on key ferroptosis-associated proteins. In both 22Rv1 and LNCaP cells, ZNF419 knockdown led to a marked reduction in GPX4 protein levels (Fig. 4A, B and Supplementary Fig. 3C and D). Notably, this decrease was not accompanied by any significant change in *GPX4* mRNA expression as determined by RT-qPCR (Fig. 4C and D), indicating that ZNF419 regulates GPX4 mainly at the post-translational level.

**Fig. 4:**
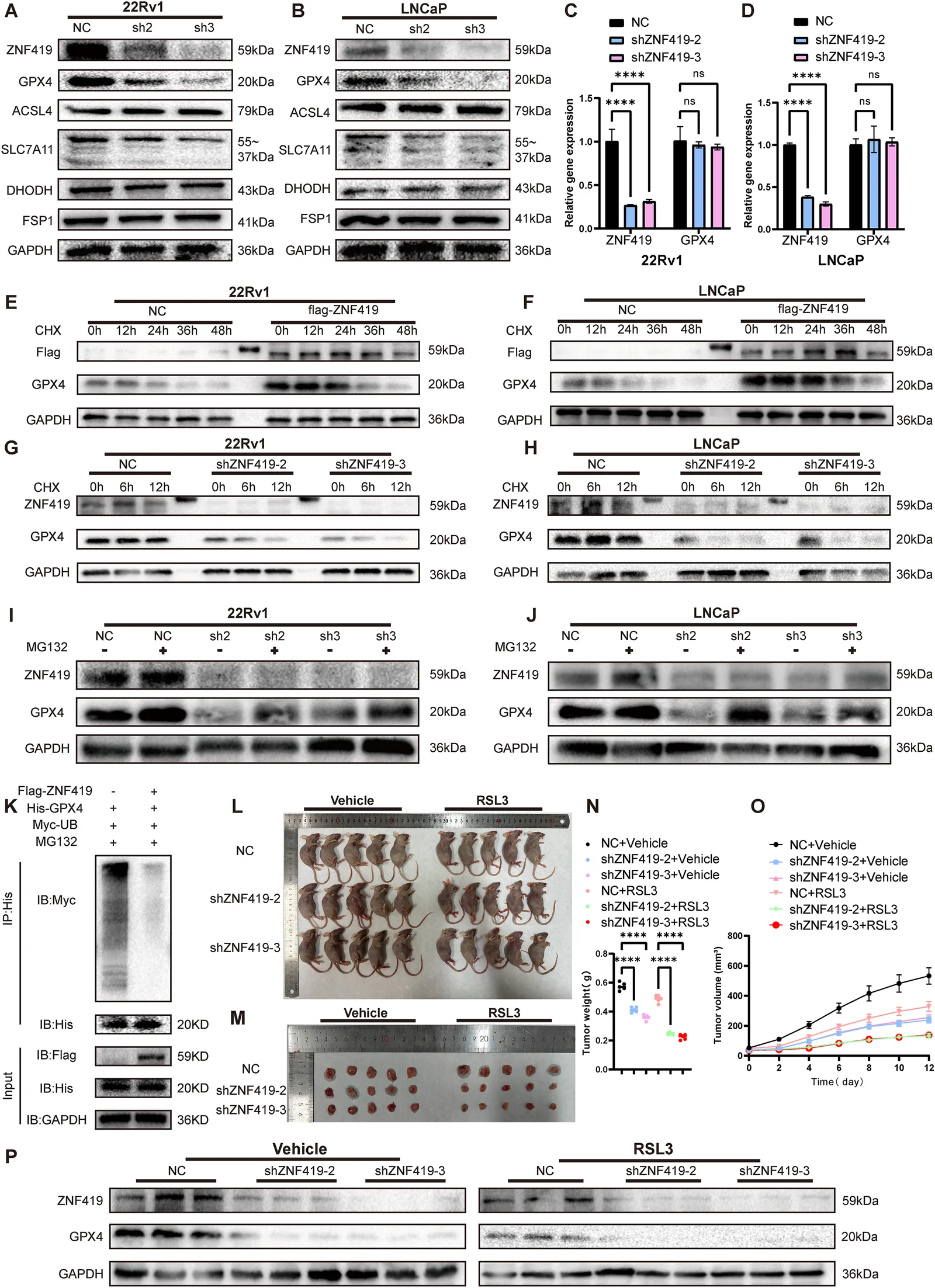
ZNF419 regulates ferroptosis sensitivity in prostate cancer cells by stabilizing GPX4 through limiting ubiquitin-mediated proteasomal degradation. (A, B) Western blot analysis of ferroptosis-associated proteins, including GPX4, ACSL4, SLC7A11, DHODH, and FSP1, in 22Rv1 and LNCaP cells transfected with control shRNA (NC) or ZNF419 shRNAs. GPX4 protein levels were decreased upon ZNF419 knockdown. (C, D) RT-qPCR analysis of *ZNF419* and *GPX4* mRNA levels in 22Rv1 and LNCaP cells following ZNF419 knockdown. GPX4 mRNA levels were not significantly altered by ZNF419 depletion. (E, F) Representative CHX-chase immunoblots showing GPX4 degradation in control and ZNF419-overexpressing 22Rv1 and LNCaP cells treated with CHX (100 μg/mL) for 0, 12, 24, 36, or 48 h. (G, H) Representative CHX-chase immunoblots showing GPX4 degradation in control and ZNF419-depleted 22Rv1 and LNCaP cells treated with CHX (100 μg/mL) for 0, 6, or 12 h. (I, J) Western blot analysis of GPX4 protein expression in ZNF419-depleted 22Rv1 and LNCaP cells treated with or without MG132 (10 μM) for 8 h before cell harvest. (K) Ubiquitination analysis of His-GPX4 in cells co-transfected with Myc-ubiquitin and Flag-ZNF419. MG132 (10 μM) was added for 8 h before cell harvest, His-GPX4 was immunoprecipitated, and ubiquitinated GPX4 was detected by anti-Myc immunoblotting. (L, M) Representative images of tumor-bearing nude mice and excised xenograft tumors derived from control or ZNF419-depleted 22Rv1 cells treated with vehicle or RSL3. RSL3 was administered intraperitoneally at 100 mg/kg every 3 days over a 12-day treatment period. The vehicle consisted of 5% DMSO, 40% PEG300, 5% Tween 80, and 50% ddH_2_O and was administered according to the same schedule. (N) Quantification of xenograft tumor weight in the indicated groups. (O) Tumor growth curves of xenografts from the indicated groups, with tumor size measured every two days during the 12-day treatment period. (P) Western blot analysis of ZNF419 and GPX4 protein expression in xenograft tumor tissues under the indicated conditions. GAPDH was used as the loading control. Data are presented as mean ± SD. In vitro quantitative data were obtained from three independent biological experiments, and xenograft experiments included five mice per group. RT-qPCR data in panels C and D were analyzed using two-way ANOVA followed by Tukey's multiple-comparisons test. Xenograft tumor weights in panel N were analyzed using ordinary one-way ANOVA followed by Tukey's multiple-comparisons test. Tumor growth curves in panel O were analyzed using two-way repeated-measures ANOVA, with time as the repeated-measures factor and treatment group as the between-subject factor. *p < 0.05, **p < 0.01, ***p < 0.001, ****p < 0.0001; ns, not significant.

To investigate whether ZNF419 influences GPX4 protein stability, we performed cycloheximide (CHX) chase assays. For quantification, GPX4 levels at each time point were normalized to the corresponding loading control and then expressed relative to the 0-h value within the same experimental group. Using this normalization approach, ZNF419 overexpression delayed the relative degradation of GPX4 protein, whereas ZNF419 depletion accelerated GPX4 degradation in both PCa cell lines (Fig. 4E–H and Supplementary Fig. 3E–H). Furthermore, inhibition of proteasomal degradation with MG132 restored GPX4 protein levels reduced by ZNF419 knockdown (Fig. 4I, J and Supplementary Fig. 3I and J), indicating that proteasome-dependent degradation contributes to ZNF419-mediated regulation of GPX4.

To directly determine whether ZNF419 regulates GPX4 ubiquitination, we performed tagged GPX4 ubiquitination assays. His-GPX4 was immunoprecipitated from cells co-transfected with Myc-ubiquitin and Flag-ZNF419, and ubiquitinated GPX4 was detected by anti-Myc immunoblotting. ZNF419 overexpression markedly reduced the ubiquitination level of GPX4, providing direct biochemical evidence that ZNF419 limits GPX4 ubiquitination (Fig. 4K). Together with the CHX chase and MG132 rescue experiments, these data support that ZNF419 stabilizes GPX4, at least in part, by suppressing ubiquitin-mediated proteasomal degradation.

In a subcutaneous xenograft model, ZNF419 knockdown was associated with reduced tumor growth, as reflected by decreased tumor volume and tumor weight compared with control groups (Fig. 4L–O). Under the experimental dosing conditions used in this study, RSL3 treatment was associated with further tumor growth inhibition in ZNF419-depleted xenografts. Western blot analysis of xenograft tissues showed reduced GPX4 expression in ZNF419-depleted tumors, with additional changes observed under RSL3 treatment (Fig. 4P). Given the limited systemic exposure and potential off-target effects of high-dose RSL3, these findings are interpreted as exploratory GPX4-proximal pharmacological evidence rather than as proof of therapeutic efficacy or definitive in vivo ferroptosis induction. Together, the data are consistent with increased vulnerability of ZNF419-depleted tumors to ferroptosis-associated pharmacological stress.

Collectively, these findings support a model in which ZNF419 contributes to GPX4 protein stability by limiting ubiquitin-mediated proteasomal degradation, thereby modulating susceptibility to ferroptosis-inducing treatments in PCa cells.

### 2.5 ZNF419 promotes *ZDHHC20* expression and is associated with activation of a candidate downstream enhancer region

To further elucidate the molecular mechanism underlying ZNF419-mediated regulation, we analyzed potential downstream targets of ZNF419 using the ChIP-seq datasets GSM2466550 [34] and GSM2466549 [34] obtained from the Cistrome DB database. Venn intersection analysis identified 75 overlapping genes, among which genes involved in post-translational modifications were prioritized. ZDHHC20 emerged as a candidate downstream effector (Fig. 5A and B). As a member of the palmitoyltransferase family, ZDHHC20 has been reported to promote GPX4 palmitoylation, thereby preventing its ubiquitination and degradation [35].

**Fig. 5:**
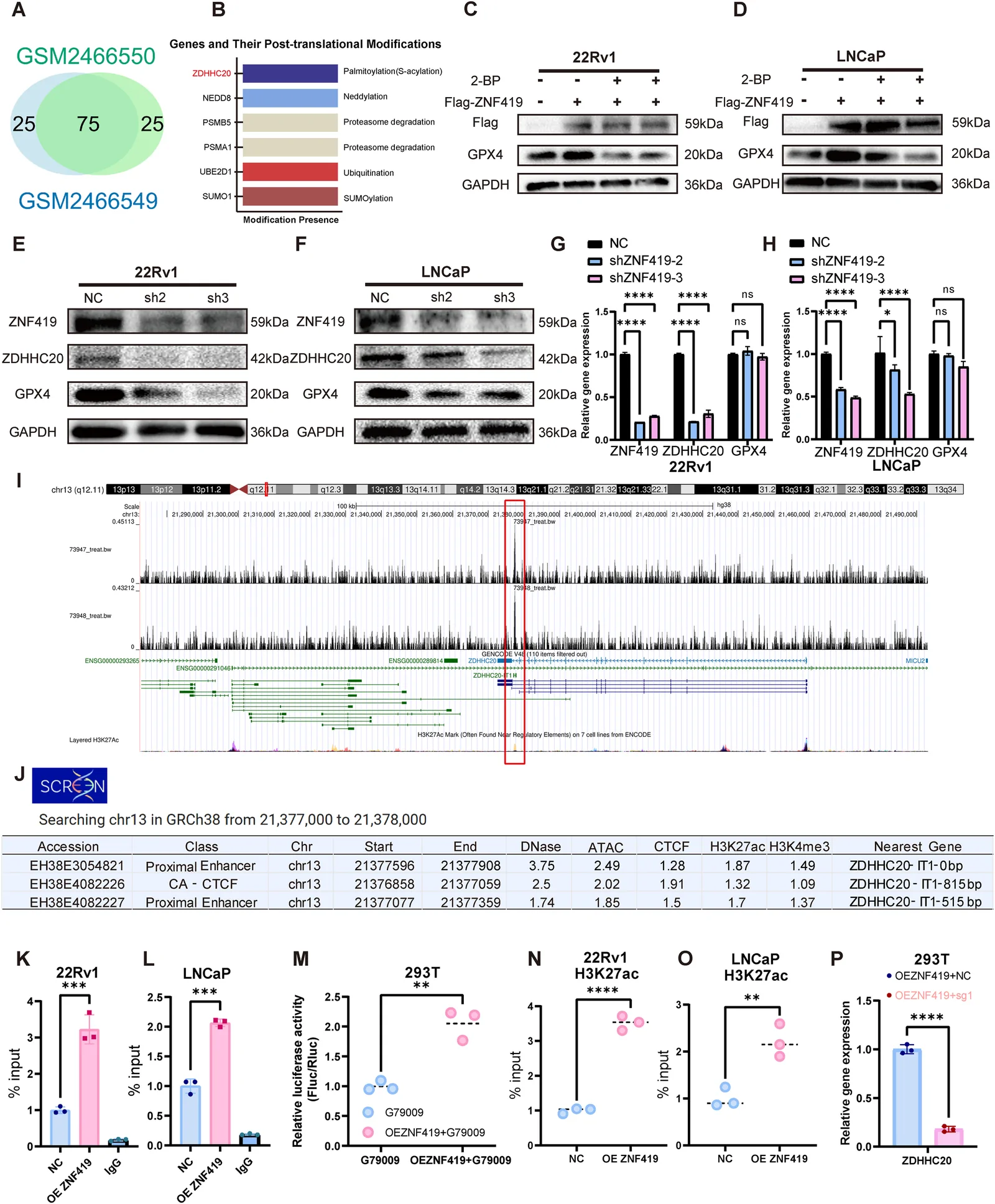
ZNF419 promotes ZDHHC20 expression and is associated with activation of a candidate downstream enhancer region. (A) Venn diagram showing overlapping genes from ZNF419 ChIP-seq datasets GSM2466550 and GSM2466549. (B) Functional categorization of candidate genes associated with post-translational modifications. (C, D) Western blot analysis of GPX4 protein expression in ZNF419-overexpressing 22Rv1 and LNCaP cells treated with or without the broad palmitoylation inhibitor 2-BP (10 μM) for 24 h. (E, F) Western blot analysis showing decreased ZDHHC20 and GPX4 protein expression upon ZNF419 knockdown in 22Rv1 and LNCaP cells. (G, H) RT-qPCR analysis showing reduced *ZDHHC2*0 mRNA expression after ZNF419 knockdown, whereas *GPX4* mRNA levels were not significantly altered. (I) UCSC Genome Browser tracks showing ZNF419 ChIP-seq peaks located downstream of the *ZDHHC20* gene. (J) SCREEN database prediction identifying this downstream region as a putative enhancer of *ZDHHC20.* (K, L) ZNF419 ChIP-qPCR analysis showing enrichment of ZNF419 at the candidate ZDHHC20 enhancer region in 22Rv1 (K) and LNCaP (L) cells following ZNF419 overexpression, with IgG included as a negative control. (M) Enhancer reporter assay showing increased reporter activity driven by the candidate ZDHHC20 enhancer region upon ZNF419 overexpression in 293T cells. (N, O) H3K27ac ChIP-qPCR analysis showing increased H3K27ac enrichment at the candidate enhancer region following ZNF419 overexpression in 22Rv1 (N) and LNCaP (O) cells. (P) RT-qPCR analysis showing that CRISPR/Cas9-mediated perturbation of the candidate ZDHHC20 enhancer region attenuated ZNF419-associated upregulation of ZDHHC20 expression in 293T cells. Data are presented as mean ± SD from three independent biological experiments. RT-qPCR data in panels G and H were analyzed using two-way ANOVA followed by Tukey's multiple-comparisons test. Quantitative data in panels K–P were analyzed using a two-tailed paired Student's t-test. *p < 0.05, **p < 0.01, ***p < 0.001, ****p < 0.0001; ns, not significant.

To examine whether palmitoylation may contribute to the ZNF419-associated regulation of GPX4, PCa cells overexpressing ZNF419 were treated with the broad palmitoylation inhibitor 2-bromopalmitate (2-BP). 2-BP attenuated the increase in GPX4 protein observed under ZNF419-overexpression conditions (Fig. 5C, D and Supplementary Fig. 4A and B), suggesting that palmitoylation may contribute to this regulatory process. Analysis of the GEPIA2 database further revealed a positive correlation between *ZNF419* and *ZDHHC20* expression (Supplementary Fig. 4C). Consistently, ZNF419 knockdown reduced ZDHHC20 protein (Fig. 5E, F and Supplementary Fig. 4D and E) and mRNA levels (Fig. 5G and H), whereas GPX4 mRNA was not significantly altered in 22Rv1 and LNCaP cells.

Examination of publicly available ZNF419 ChIP-seq data using the UCSC Genome Browser identified a ZNF419 occupancy signal at a genomic region located downstream of the 3′ end of the *ZDHHC20* gene, rather than at its promoter (Fig. 5I). Further analysis using the SCREEN database identified this downstream region as a putative enhancer of *ZDHHC20* (Fig. 5J).

ZNF419 ChIP-qPCR performed in 22Rv1 and LNCaP cells showed enrichment of ZNF419 at this candidate enhancer region, with low IgG background (Fig. 5K and L). Moreover, enhancer reporter assays performed in 293T cells showed that ZNF419 overexpression increased reporter activity driven by the candidate ZDHHC20 enhancer region (Fig. 5M). H3K27ac ChIP-qPCR further showed increased H3K27ac enrichment at this candidate enhancer region following ZNF419 overexpression in both 22Rv1 and LNCaP cells (Fig. 5N and O).

To complement these endogenous chromatin-based observations with functional perturbation evidence, CRISPR/Cas9-mediated targeting of the candidate enhancer region was performed in 293T cells, which were selected because of their high transfection and genome-editing efficiency. Genomic DNA PCR/qPCR analysis showed a marked reduction in the relative genomic DNA signal at the targeted locus in enhancer-targeting sgRNA-treated cells compared with control cells, supporting perturbation of the intended enhancer region (Supplementary Fig. 4F). Consistently, perturbation of this candidate enhancer region attenuated ZNF419-associated upregulation of ZDHHC20 expression in 293T cells (Fig. 5P).

Collectively, these findings support a model in which ZNF419 promotes ZDHHC20 expression, at least in part, through a candidate downstream enhancer region. The endogenous chromatin-based evidence in prostate cancer cells is complemented by reporter and enhancer-perturbation assays performed in 293T cells.

### 2.6 ZNF419–ZDHHC20–GPX4 axis modulates ferroptosis sensitivity in PCa

To elucidate the functional consequence of the ZNF419–ZDHHC20–GPX4 regulatory cascade, we assessed GPX4 palmitoylation status using acyl-biotin exchange (ABE) assays. ZNF419 knockdown markedly reduced GPX4 palmitoylation in both 22Rv1 (Fig. 6A) and LNCaP (Fig. 6B) cells, supporting an association between ZNF419 expression, GPX4 palmitoylation status, and GPX4 stability.

**Fig. 6:**
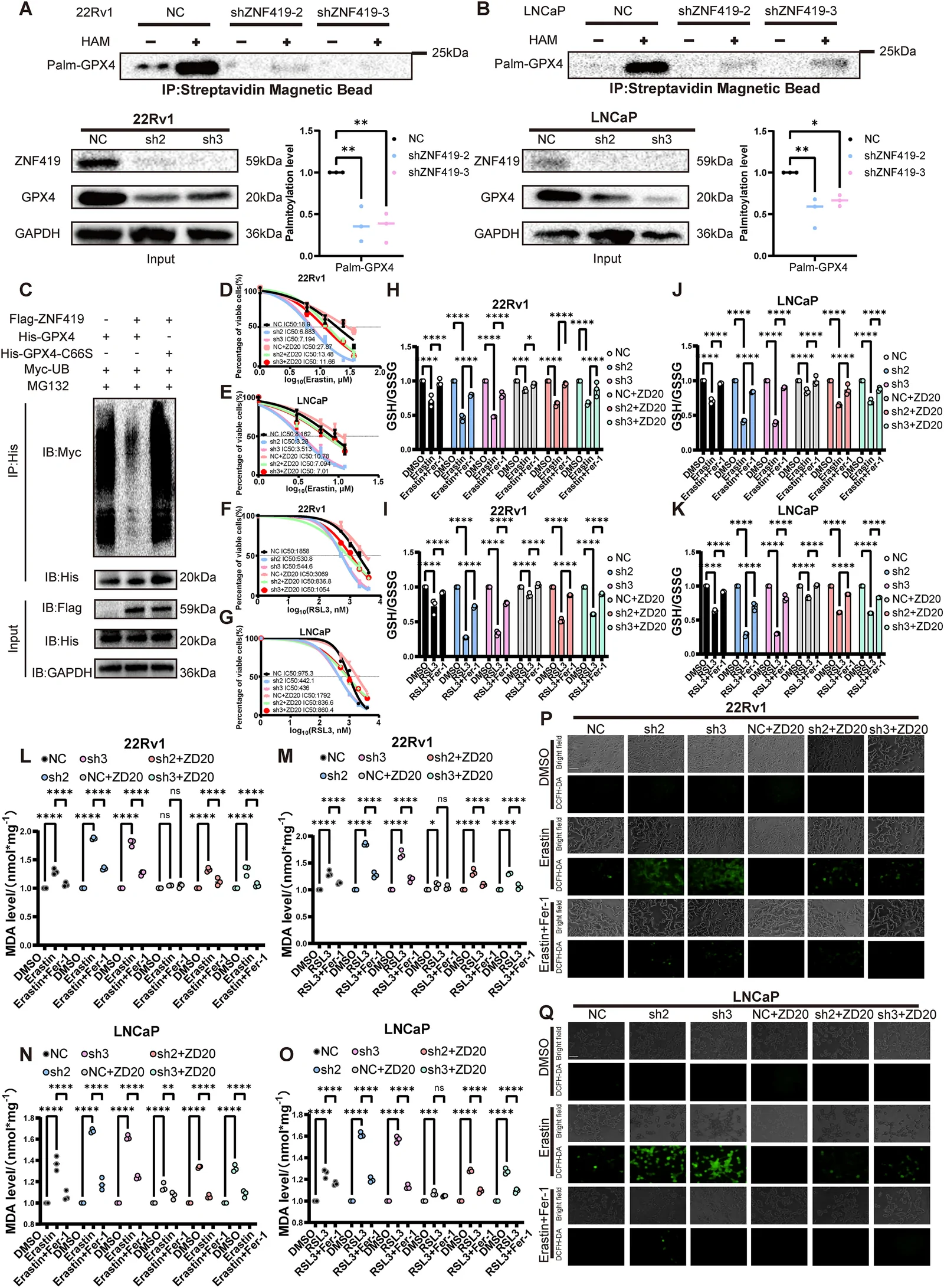
The ZNF419–ZDHHC20 axis modulates ferroptosis sensitivity in prostate cancer cells through palmitoylation-associated regulation of GPX4. (A, B) Acyl-biotin exchange (ABE) assays showing reduced GPX4 palmitoylation following ZNF419 knockdown in 22Rv1 (A) and LNCaP (B) cells. Palmitoylated GPX4 levels were quantified from three independent biological experiments. (C) Tagged ubiquitination assay comparing the ubiquitination of wild-type GPX4 and the palmitoylation-deficient GPX4-C66S mutant. HEK293T cells were transfected with Flag-ZNF419, Myc-ubiquitin, and His-GPX4 or His-GPX4-C66S constructs as indicated. His-tagged GPX4 was immunoprecipitated, and ubiquitinated GPX4 was detected by anti-Myc immunoblotting. (D, E) Dose–response curves showing erastin sensitivity in control cells, ZNF419-knockdown cells, and the corresponding groups with ZDHHC20 overexpression in 22Rv1 (D) and LNCaP (E) cells. Cells were treated with erastin at 0, 6, 12, 24, or 36 μM for 24 h. (F, G) Dose–response curves showing RSL3 sensitivity in control cells, ZNF419-knockdown cells, and the corresponding groups with ZDHHC20 overexpression in 22Rv1 (F) and LNCaP (G) cells. Cells were treated with RSL3 at 0, 0.5, 1, 2, or 4 μM for 24 h. (H, I) Intracellular GSH/GSSG ratios in 22Rv1 cells under the indicated treatment conditions. Panel H shows cells treated with erastin (10 μM), with or without Fer-1 (10 μM), for 24 h, whereas panel I shows cells treated with RSL3 (1 μM), with or without Fer-1 (10 μM), for 24 h. (J, K) Intracellular GSH/GSSG ratios in LNCaP cells under the indicated treatment conditions. Panel J shows cells treated with erastin (10 μM), with or without Fer-1 (10 μM), for 24 h, whereas panel K shows cells treated with RSL3 (1 μM), with or without Fer-1 (10 μM), for 24 h. (L, M) Malondialdehyde (MDA) levels in 22Rv1 cells under the indicated treatment conditions. Panel L shows cells treated with erastin (10 μM), with or without Fer-1 (10 μM), for 24 h, whereas panel M shows cells treated with RSL3 (1 μM), with or without Fer-1 (10 μM), for 24 h. (N, O) MDA levels in LNCaP cells under the indicated treatment conditions. Panel N shows cells treated with erastin (10 μM), with or without Fer-1 (10 μM), for 24 h, whereas panel O shows cells treated with RSL3 (1 μM), with or without Fer-1 (10 μM), for 24 h. Individual data points in panels H–O represent independent biological experiments. (P, Q) Representative fluorescence microscopy images of DCFH-DA staining showing ROS accumulation following treatment with erastin (10 μM), with or without Fer-1 (10 μM), for 24 h in 22Rv1 (P) and LNCaP (Q) cells. The indicated groups include control cells, ZNF419-knockdown cells, and the corresponding groups with ZDHHC20 overexpression. Data are presented as mean ± SD from three independent biological experiments. Quantification of GPX4 palmitoylation in panels A and B was analyzed using one-way ANOVA followed by Tukey's multiple-comparisons test. Dose–response curves in panels D–G were fitted using nonlinear regression to estimate IC50 values. Biochemical data in panels H–O were analyzed using two-way ANOVA followed by Tukey's multiple-comparisons test. Panels P and Q show representative images from three independent biological experiments. *p < 0.05, **p < 0.01, ***p < 0.001, ****p < 0.0001; ns, not significant.

To further determine whether GPX4 palmitoylation contributes to ZNF419-mediated regulation of GPX4 ubiquitination, we generated a palmitoylation-deficient GPX4 C66S mutant. In tagged ubiquitination assays, ZNF419 overexpression reduced the ubiquitination of wild-type GPX4. However, when the C66 palmitoylation site was disrupted, the ability of ZNF419 to suppress GPX4 ubiquitination was markedly attenuated, and GPX4-C66S retained a higher ubiquitination level despite ZNF419 overexpression (Fig. 6C). These results suggest that C66-dependent GPX4 palmitoylation contributes to ZNF419-mediated suppression of GPX4 ubiquitination.

Functionally, depletion of ZNF419 increased the sensitivity of PCa cells to the ferroptosis inducers erastin and RSL3, as reflected by reduced IC50 values (Fig. 6D–G). These results suggest that ZNF419 depletion increases susceptibility to treatments that induce ferroptosis-associated stress rather than causing extensive spontaneous cell death, consistent with a sensitized state that becomes evident under ferroptosis-inducing stress. Importantly, ectopic restoration of ZDHHC20 expression partially reversed the heightened sensitivity of ZNF419-depleted cells to these treatments, supporting a downstream role for ZDHHC20 in ZNF419-associated regulation of ferroptosis susceptibility.

Biochemical assays showed that ZNF419 knockdown decreased the GSH/GSSG ratio and increased MDA levels, indicating enhanced oxidative and redox alterations under ferroptosis-inducing conditions. Ectopic expression of ZDHHC20 partially restored the GSH/GSSG balance and reduced MDA accumulation, supporting that ZDHHC20 alleviates the ferroptosis-associated biochemical changes induced by ZNF419 depletion (Fig. 6H–O). Consistently, DCFH-DA-based ROS detection showed increased ROS accumulation after ZNF419 depletion under ferroptosis-inducing conditions, whereas restoration of ZDHHC20 expression attenuated this oxidative phenotype. Representative fluorescence images following erastin treatment are shown in Fig. 6P and Q, with the corresponding quantitative analysis of corrected DCF fluorescence intensity shown in Supplementary Fig. 4I and J. Similarly, representative images following RSL3 treatment are shown in Supplementary Fig. 4G and H, with the corresponding fluorescence quantification shown in Supplementary Fig. 4K and L.

Together, these findings support a model in which the ZNF419–ZDHHC20 axis contributes to GPX4 stabilization, at least in part, through palmitoylation-associated suppression of GPX4 ubiquitination, thereby reducing ferroptosis-associated oxidative stress and limiting sensitivity to ferroptosis-inducing treatments in PCa cells.

### 2.7 ZNF419 depletion and IKE treatment reduce xenograft growth in vivo, with preliminary protein-level observations in clinical specimens

To further evaluate the in vivo relevance of the ZNF419–ZDHHC20–GPX4 pathway and to reduce reliance on systemic RSL3 administration, we performed an independent xenograft experiment using imidazole ketone erastin (IKE). Mice bearing control or ZNF419-depleted xenografts were assigned to six treatment groups: NC + vehicle, NC + IKE, shZNF419-3 + vehicle, shZNF419-3 + IKE, NC + enzalutamide (ENZ), and NC + ENZ + IKE. Tukey-adjusted pairwise comparisons showed lower endpoint tumor weights and volumes in the NC + IKE, shZNF419-3 + vehicle, and NC + ENZ groups than in the NC + vehicle group. Both endpoints were also lower in the shZNF419-3 + IKE group than in the shZNF419-3 + vehicle and NC + IKE groups, and in the NC + ENZ + IKE group than in the NC + ENZ and NC + IKE groups under the tested conditions (adjusted p < 0.05 for the indicated comparisons; Fig. 7C and D). Body weights showed an overall increasing trend during the 20-day treatment period, and no obvious treatment-associated body-weight loss was observed among the six experimental groups (Fig. 7E). However, because shZNF419-3 + ENZ and shZNF419-3 + ENZ + IKE groups were not included, this experiment was not designed to evaluate a complete three-factor interaction among ZNF419 depletion, ENZ, and IKE.

**Fig. 7:**
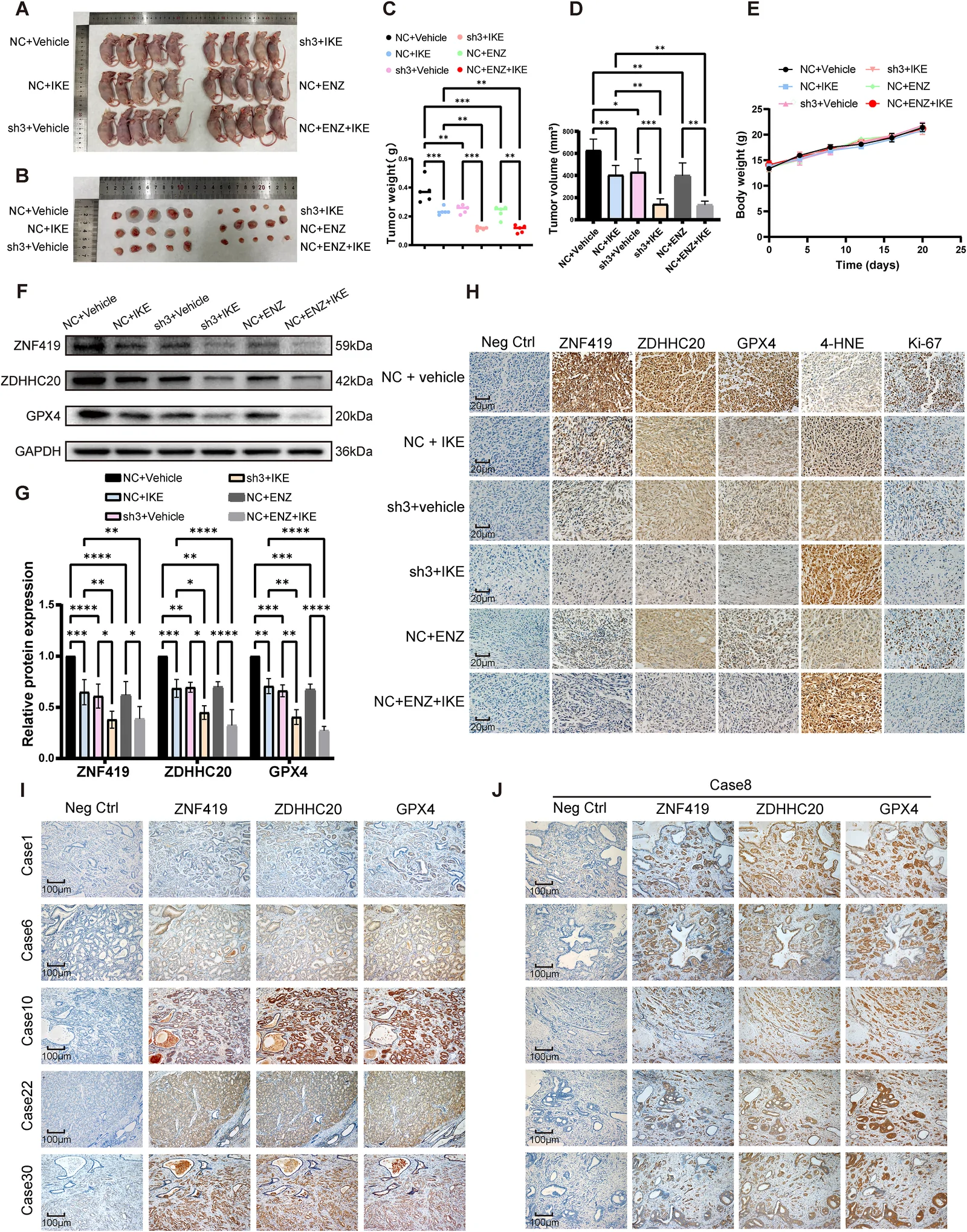
ZNF419 depletion and IKE treatment reduce xenograft growth in vivo, with elevated expression of ZNF419, ZDHHC20, and GPX4 in clinical prostate cancer specimens. (A, B) Representative images of tumor-bearing mice (A) and excised xenograft tumors (B) from the indicated treatment groups, including NC + vehicle, NC + IKE, shZNF419-3 + vehicle, shZNF419-3 + IKE, NC + enzalutamide (ENZ), and NC + ENZ + IKE. IKE was administered intraperitoneally at 40 mg/kg every 4 days over a 20-day treatment period, whereas ENZ was administered at 10 mg/kg/day by oral gavage throughout the same treatment period. (C) Tumor weights of xenografts from the indicated groups at the experimental endpoint. (D) Tumor volumes of xenografts from the indicated groups at the experimental endpoint. (E) Body weights of mice in the NC + Vehicle, NC + IKE, shZNF419-3 + Vehicle, shZNF419-3 + IKE, NC + ENZ, and NC + ENZ + IKE groups were monitored during the 20-day treatment period. Data are presented as mean ± SD; n = 5 mice per group. (F) Western blot analysis of ZNF419, ZDHHC20, and GPX4 protein expression in xenograft tumor tissues from the indicated groups. GAPDH was used as a loading control. (G) Quantification of ZNF419, ZDHHC20, and GPX4 protein expression in xenograft tumor tissues. Protein levels were normalized to GAPDH. (H) Representative immunohistochemical staining images of xenograft tumor tissues from the indicated groups. Negative controls were prepared by omission of the primary antibody. Reduced ZNF419 staining in the shZNF419-3 xenografts was observed relative to the corresponding NC groups, consistent with the reduction in ZNF419 protein abundance shown by Western blotting in panel F. (I) Representative IHC staining of ZNF419, ZDHHC20, and GPX4 in human prostate cancer cases with variable ZNF419 expression levels. From top to bottom, representative cases show low ZNF419 expression in a Gleason pattern 3 region, moderate ZNF419 expression in a Gleason pattern 3 region, high ZNF419 expression in a Gleason pattern 3 region, moderate ZNF419 expression in a Gleason pattern 4 region, and moderately strong ZNF419 expression in a mixed Gleason pattern 3 + 5 region. The images illustrate the range of staining intensities and are presented descriptively; patient-level associations were evaluated using the complete continuous mean H-score dataset in Supplementary Table 1 and Supplementary Fig. 6. Negative-control staining was performed by omission of the primary antibody. Scale bars are indicated in the images. (J) Representative IHC staining of ZNF419, ZDHHC20, and GPX4 in different histological regions from the same patient specimen. From top to bottom, the regions correspond to Gleason 3 + 3, Gleason 3 + 3, Gleason 3 + 5, Gleason 4 + 5 with a minor Gleason pattern 3 component, and Gleason 4 + 5 with partial Gleason pattern 3 components. ZNF419 staining did not show an obvious Gleason pattern-dependent increase across these representative regions. These images are descriptive and are not used to establish a grade-dependent relationship. Scale bars are indicated in the images. Data are presented as mean ± SD. Xenograft experiments included five mice per group. Endpoint tumor weights in panel C and endpoint tumor volumes in panel D were analyzed separately using ordinary one-way ANOVA followed by Tukey's multiple-comparisons test encompassing all 15 pairwise comparisons among the six experimental groups for each endpoint. Brackets highlight seven comparisons relevant to the experimental questions, including comparisons of individual interventions with vehicle and of combined interventions with the corresponding individual interventions. Multiple-comparison adjustment included all 15 pairwise comparisons for each endpoint, irrespective of which comparisons are annotated. Western blot quantification in panel G was analyzed separately for each protein using ordinary one-way ANOVA followed by Tukey's multiple-comparisons test. *p < 0.05, **p < 0.01, ***p < 0.001, ****p < 0.0001; ns, not significant.

Western blot analysis of xenograft tumor tissues showed that ZNF419 depletion was accompanied by reduced ZDHHC20 and GPX4 protein expression, and IKE treatment further decreased pathway-related protein levels in the indicated groups (Fig. 7F and G). Representative immunohistochemical staining of xenograft tumor tissues further supported pathway-related changes after ZNF419 depletion and IKE treatment (Fig. 7H). These findings provide supportive in vivo evidence of lower tumor burden with combined ZNF419 depletion and IKE treatment than with either intervention alone under the tested conditions. The earlier enzalutamide-focused xenograft experiment using systemic RSL3 is provided as supplementary exploratory evidence because of the pharmacokinetic and interpretive limitations of systemic RSL3 administration (Supplementary Fig. 5).

To examine the protein-level expression of ZNF419, ZDHHC20, and GPX4 in human prostate cancer specimens, we performed immunohistochemical staining in 30 paired human prostate cancer and adjacent non-tumor tissues. Negative controls were prepared by omission of the primary antibody. Representative tumor specimens showed variable staining intensities for the three proteins (Fig. 7I and J). For each marker and tissue, the mean of the H-scores independently assigned by two pathologists was analyzed as a continuous measure and was also converted into a predefined ordinal staining category. Both the continuous mean H-scores and the ordinal categories were significantly higher in prostate cancer tissues than in paired adjacent non-tumor tissues (Supplementary Table 1). Overall categorical concordance between the two pathologists was high: 163 of 180 marker–specimen assessments (90.6%) were assigned to the same category, whereas 17 (9.4%) differed by one category and none differed by more than one category. In tumor tissues, continuous mean H-scores showed a positive correlation between ZNF419 and ZDHHC20 (Spearman ρ = 0.457, p = 0.011; Benjamini–Hochberg-adjusted p = 0.033). The correlation between ZNF419 and GPX4 was not statistically significant (ρ = 0.264, p = 0.159; adjusted p = 0.159), and the nominal association between ZDHHC20 and GPX4 (ρ = 0.373, p = 0.042) did not remain statistically significant after correction for the three pairwise comparisons (adjusted p = 0.064) (Supplementary Table 1 and Supplementary Fig. 6). Pairwise correlations based on the ordinal categories did not reach statistical significance. Neither analytical approach established a robust or consistent relationship with ISUP grade group or Gleason score. A nominal difference in continuous GPX4 H-score across ISUP groups (Kruskal–Wallis p = 0.020) did not remain statistically significant after correction across the three markers (adjusted p = 0.060), and GPX4 showed no monotonic correlation with ISUP grade group (ρ = −0.083, p = 0.662). These findings provide preliminary protein-level evidence that ZNF419, ZDHHC20, and GPX4 staining is elevated in prostate cancer tissues, without establishing coordinated pathway activity, biomarker utility, or a grade-dependent expression pattern.

Collectively, our results support a model in which DHT-activated AR signaling increases ZNF419 expression, and ZNF419 subsequently promotes ZDHHC20 expression through a candidate downstream enhancer region. The ZNF419–ZDHHC20 axis contributes to GPX4 palmitoylation-associated stabilization, thereby helping maintain lipid redox balance and reducing susceptibility to ferroptosis-inducing stress. Conversely, enzalutamide-mediated AR inhibition disrupts this pathway, leading to reduced ZNF419/ZDHHC20 expression, GPX4 destabilization, and increased ferroptosis-associated vulnerability. Together, these findings support an AR-associated ZNF419–ZDHHC20–GPX4 regulatory axis that modulates ferroptosis sensitivity in prostate cancer.

## 3 Discussion

Ferroptosis has emerged as an important form of regulated cell death in cancer biology, characterized by excessive lipid peroxidation and iron-dependent oxidative stress. Despite accumulating evidence implicating ferroptosis in PCa progression and therapeutic resistance, the upstream regulatory mechanisms that connect AR signaling to ferroptosis regulation remain incompletely understood. In this study, we identify ZNF419 as a previously unrecognized AR-regulated zinc finger protein associated with reduced ferroptosis susceptibility, at least in part through the ZDHHC20–GPX4 pathway, thereby linking androgen signaling to lipid redox regulation in PCa.

Mechanistically, our data support that AR is enriched at predicted ARE-containing elements within the promoter-proximal region of ZNF419, thereby contributing to transcriptional activation of ZNF419 expression. We therefore interpret ZNF419 as an AR-regulated gene rather than a definitively validated direct AR target, because the contribution of distal AR-bound regulatory elements and cooperating transcriptional mechanisms has not been fully dissected. Although our data support a functional role of promoter-proximal AR binding in ZNF419 activation, we acknowledge that distal AR-bound regulatory elements identified by ChIP-seq may also contribute to ZNF419 regulation. The relative contribution of these distal elements remains to be determined by future studies using chromatin-conformation capture, CRISPR-mediated enhancer deletion/interference, or enhancer reporter assays. This regulation is functionally relevant: androgen stimulation increased ZNF419 expression in AR-positive prostate cancer cells, whereas AR knockdown or pharmacologic AR blockade with enzalutamide reduced ZNF419 expression and was associated with GPX4 destabilization and increased ferroptosis-associated vulnerability. These findings support ZNF419 as an AR-regulated upstream component of the ZNF419–ZDHHC20–GPX4 pathway.

Functionally, ZNF419 depletion increased cellular sensitivity to erastin and RSL3, while ferrostatin-1 partially restored cell viability, supporting the involvement of a ferroptosis-associated component in the observed cytotoxic response. The accompanying increases in general ROS and MDA levels and the reduction in the GSH/GSSG ratio provide additional evidence of oxidative and redox disruption. However, DCFH-DA is a general ROS probe, whereas MDA and GSH/GSSG are supportive but not lipid peroxidation-specific readouts. Because lipid peroxidation-specific assays, such as C11-BODIPY 581/591 staining or oxidized phospholipid profiling, were not performed, the present data should not be interpreted as definitive evidence of ferroptotic lipid peroxidation or ferroptotic cell death. Rather, these findings support the conclusion that the ZNF419–ZDHHC20–GPX4 pathway modulates vulnerability to ferroptosis-associated stress. Future studies incorporating lipid peroxidation-specific assays will be required to further define the extent and specificity of ferroptotic lipid oxidation in this model.

At the post-translational level, our data support a model in which ZNF419 contributes to GPX4 protein stability, at least in part, through regulation of ZDHHC20, a palmitoyltransferase previously implicated in GPX4 palmitoylation. The tagged GPX4 ubiquitination assays showed that ZNF419 overexpression reduced GPX4 ubiquitination, providing biochemical support for the CHX-chase and MG132 rescue data. In addition, the GPX4-C66S mutant experiment showed that substitution of the C66 residue attenuated the ability of ZNF419 to suppress GPX4 ubiquitination, supporting the involvement of C66-dependent, palmitoylation-associated regulation in ZNF419-mediated protection of GPX4 from ubiquitin-mediated proteasomal degradation. 2-BP is a broad palmitoylation inhibitor that may affect other lipid modifications; the 2-BP results were therefore interpreted in the context of complementary ABE assays and GPX4-C66S mutant experiments rather than as standalone evidence. The present data do not definitively establish that ZDHHC20 directly catalyzes GPX4 palmitoylation in our experimental system. Future studies using catalytically inactive ZDHHC20 mutants and in vitro palmitoylation assays will be required to confirm this biochemical step. Although GPX4 is a selenoprotein, our current data primarily support a post-translational mechanism involving GPX4 palmitoylation, ubiquitination, and proteasomal degradation. Nevertheless, we cannot exclude the possibility that ZNF419 may also affect other selenoproteins or selenium-related pathways. Systematic evaluation of additional selenoproteins will therefore be required in future studies to determine whether ZNF419 has broader effects on selenoprotein availability. The ZNF419 overexpression experiments were used as mechanistic gain-of-function assays and may not fully reflect the physiological range of ZNF419 expression in clinical tumors. Therefore, these data were interpreted together with knockdown, endogenous AR-regulatory evidence, and downstream ZDHHC20 rescue experiments rather than as standalone evidence.

This mechanistic cascade also provides new insight into how enzalutamide may exert antitumor activity beyond canonical AR blockade. By downregulating *ZNF419*, enzalutamide may impair ZDHHC20-mediated GPX4 stabilization, thereby increasing ferroptosis-associated vulnerability. These findings complement previous observations linking altered ferroptosis susceptibility to enzalutamide response and provide a rationale for further investigating whether modulation of ferroptosis-associated vulnerability may influence the efficacy of AR-targeted therapy. In this context, combined targeting of AR signaling and ferroptosis-associated defense pathways, including GPX4-centered regulatory mechanisms, warrants further preclinical investigation as a potential approach to modulating treatment response in advanced prostate cancer.

The in vivo RSL3 experiments should be interpreted cautiously. RSL3 is not a clinically or pharmacokinetically optimized GPX4 inhibitor, and its limited systemic exposure, formulation constraints, and potential off-target effects at high doses preclude interpretation of the present dosing regimen as a clinically translatable therapeutic strategy. In this study, RSL3 was used as a short-term exploratory pharmacological tool to challenge the GPX4-proximal arm of the pathway, because the central mechanism under investigation involves ZDHHC20-associated, palmitoylation-related regulation of GPX4 stability. Accordingly, the RSL3 xenograft findings are interpreted as supportive GPX4-proximal pharmacological evidence rather than as definitive proof of in vivo ferroptosis induction or therapeutic efficacy. The observed tumor-growth effects should therefore be considered together with the broader mechanistic evidence, including GPX4 destabilization, ubiquitination and palmitoylation changes, ferrostatin-1-sensitive responses, and ZDHHC20 rescue experiments.

The six-group IKE xenograft experiment included five mice per group, and no formal a priori power calculation was performed. The relatively small group size limits the precision of the effect estimates and may reduce sensitivity to smaller between-group differences after multiple-comparison correction. These findings therefore provide exploratory in vivo support and require confirmation in larger studies with prospectively specified comparisons and sample sizes justified by a priori power calculations. Furthermore, the observed pairwise differences alone do not establish statistical interaction or pharmacological synergy between the interventions.

Our clinical IHC analysis provides preliminary protein-level evidence that ZNF419, ZDHHC20, and GPX4 staining is elevated in human prostate cancer tissues. Both continuous mean H-scores and H-score-derived ordinal categories were significantly higher in tumor tissues than in paired adjacent non-tumor tissues, and 25 of the 30 tumor specimens showed moderate-to-strong staining for all three markers. Continuous mean H-score analysis identified a positive ZNF419–ZDHHC20 correlation that remained significant after correction for the three pairwise comparisons, whereas the ZNF419–GPX4 correlation was not significant and the nominal ZDHHC20–GPX4 association did not remain significant after correction. No robust or consistent association with ISUP grade group or Gleason score was established. These observations do not demonstrate coordinated activation of the complete pathway or prognostic, predictive, or treatment-response biomarker value. Lower inter-observer agreement for ZNF419 in adjacent non-tumor tissues also limits the interpretation of fine quantitative differences in low-level staining (Supplementary Table 1). For ZNF419 specifically, concordance between genetic depletion, reduced protein abundance in xenograft lysates, and reduced staining in the corresponding FFPE xenograft tissues provides orthogonal support for target-associated IHC staining under the conditions used. Nevertheless, dedicated knockdown/overexpression FFPE cell-pellet controls or validation with independent antibodies would further strengthen specificity assessment, particularly for ZDHHC20 and GPX4. Larger clinically annotated cohorts with AR-status, outcome, and treatment-response information will be required to define the clinical significance of these proteins.

Interestingly, ZNF419 expression was also detectable in AR-negative PCa cells (Fig. 1H and Supplementary Fig. 1C), suggesting that its aberrant activation is not strictly dependent on AR and may be driven by alternative signaling mechanisms. Short-term DHT stimulation (6 h) increased ZNF419 protein levels (Supplementary Fig. 4M and N), raising the possibility that rapid AR-associated signaling mechanisms may also contribute to ZNF419 regulation [36,37]. Our data suggest that ERK activation may contribute to DHT-associated upregulation of ZNF419, but this requires further mechanistic validation. The DHT-associated increase in ZNF419 was attenuated by U0126 (Supplementary Fig. 4O and P), consistent with prior reports that AR can modulate ERK signaling [38]. Notably, ERK hyperactivation is frequently observed in enzalutamide-resistant or AR-deficient PCa, and our data further demonstrate that U0126 treatment markedly downregulated ZNF419 in AR-null PC3 and DU145 cell lines (Supplementary Fig. 4Q and R). Together, these findings raise the possibility that ERK signaling may contribute to ZNF419 expression in AR-low or AR-negative contexts. This possibility requires further functional validation and should be investigated in future studies.

In addition, ZNF419 knockdown not only reduced GPX4 protein levels but also led to a visible decrease in SLC7A11 (xCT), the light chain subunit of the system Xc− cystine/glutamate antiporter (Fig. 4A and B). Subsequent RT-qPCR analysis confirmed that SLC7A11 mRNA expression was reduced following ZNF419 silencing (Supplementary Fig. 4S and T). This observation is consistent with previous findings showing that AR-FL and AR-Vs can transcriptionally activate SLC7A11 [23]. The precise mechanism by which ZNF419 modulates SLC7A11 transcription remains to be elucidated; this reduction should therefore be regarded as a supportive observation rather than evidence that AR regulates SLC7A11 through ZNF419. In addition, ZNF419 knockdown reduced GPX4 protein expression in AR-negative PC3 and DU145 cells (Supplementary Fig. 4U and V), suggesting that the downstream ZNF419–GPX4 regulatory relationship may not be strictly limited to AR-positive contexts. However, because comprehensive ferroptosis sensitivity assays, CHX chase assays, and rescue experiments were not performed in these AR-negative models, whether ZNF419 controls GPX4 stability and ferroptosis sensitivity through the same ZDHHC20-dependent mechanism in AR-negative prostate cancer cells remains to be determined.

A further limitation is that an shRNA-resistant ZNF419 re-expression rescue experiment was not performed. Although two independent ZNF419 shRNAs produced concordant phenotypes, reciprocal ZNF419 overexpression yielded opposite effects, and downstream ZDHHC20 restoration partially reversed the observed phenotypes, these complementary approaches reduce but do not completely eliminate the possibility of shRNA-related off-target effects. Therefore, the absence of a direct shRNA-resistant ZNF419 rescue remains an important limitation of the present study and requires further genetic validation. Future studies using shRNA-resistant ZNF419 rescue or orthogonal genome-editing strategies will be important to further validate the proposed pathway.

In summary, this study supports a previously unrecognized AR-associated regulatory model in which the ZNF419–ZDHHC20–GPX4 pathway modulates ferroptosis-associated vulnerability in PCa. Through coordinated transcriptional and post-translational regulation, ZNF419 contributes to GPX4 stabilization and cellular redox homeostasis, thereby reducing susceptibility to ferroptosis-inducing stress in PCa cells. These findings expand the conceptual framework of androgen-associated ferroptosis regulation and provide a rationale for further investigating whether modulation of this pathway may influence responses to AR-targeted therapy.

## 4 Materials and methods

### 4.1 Materials

RSL3 (S8155), enzalutamide (MDV3100, S1250), erastin (S7242), ferrostatin-1 (Fer-1; S7243), Nec-1 (S8037), Z-VAD-FMK (S7023), and imidazole ketone erastin (IKE; S8877) were purchased from Selleck Chemicals. GSH and GSSG Assay Kit (S0053), Lipid Peroxidation MDA Assay Kit (S0131S), and Reactive Oxygen Species Assay Kit (S0033S) were obtained from Beyotime. Antibodies were purchased from the following sources: ZNF419 (Proteintech, 25724-1-AP), SLC7A11 (Proteintech, 26864-1-AP), GPX4 (Proteintech, 67763-1-Ig), ACSL4 (Proteintech, 22401-1-AP), FSP1 (Proteintech, 20886-1-AP), DHODH (Proteintech, 14877-1-AP), 4-HNE (Proteintech, 68538-1-Ig), ZDHHC20 (Solarbio, K111807P), GAPDH (Proteintech, 60004-1-Ig), AR (Santa Cruz Biotechnology, sc-7305).

### 4.2 Cell culture

The human PCa cell lines 22Rv1, LNCaP, PC3, and DU145, as well as the human embryonic kidney cell line 293T, were obtained from Procell Life Science & Technology Co., Ltd. LNCaP, 22Rv1, and PC3 cells were cultured in RPMI 1640 medium (Shanghai D&R, Cat# DT31800) supplemented with 10% fetal bovine serum (FBS; Shanghai D&R, Cat# DT-500-S). DU145 cells were cultured in Minimum Essential Medium (MEM; Procell, Cat# PM150410) supplemented with 10% FBS and non-essential amino acids (NEAA). 293T cells were cultured in Dulbecco's Modified Eagle Medium (DMEM; Cat# PM150210) containing 10% FBS. All cells were maintained at 37°C in a humidified atmosphere with 5% CO_2_. The identities of the cell lines were verified based on authentication records provided by the corresponding cell banks or suppliers. Cells were routinely tested for mycoplasma contamination, and only mycoplasma-negative cultures were used for experiments.

### 4.3 Patients and clinical specimens

This study was approved by the Institutional Review Board of China Medical University. Written informed consent was obtained from all participants, and all procedures were conducted in accordance with the principles of the Declaration of Helsinki. Thirty paired prostate cancer and adjacent non-tumor tissue specimens were obtained from 30 patients who underwent surgical resection at the First Hospital of China Medical University between 2018 and 2025. All patients had a histopathological diagnosis of prostate adenocarcinoma and had not received radiotherapy or chemotherapy prior to surgery.

### 4.4 Intracellular ROS detection

Intracellular ROS levels were measured using the DCFH-DA fluorescent probe with a ROS assay kit (Beyotime, Cat# S0033S). DCFH-DA was diluted in serum-free culture medium at a ratio of 1:1000 to a final concentration of 10 μmol/L. After removing the culture medium, 1 mL of the diluted DCFH-DA solution was added to each well, followed by incubation at 37°C for 20 min. After incubation, cells were washed three times with serum-free medium to remove excess probe. The fluorescence was then observed and imaged using a fluorescence inverted microscope. For each treatment group within each independent biological experiment, three non-overlapping fields were selected from spatially separated regions of each well, without preferential selection based on fluorescence intensity. Fluorescence images were acquired using identical imaging settings across treatment groups within each independent biological experiment, and the same imaging settings were maintained across the three independent biological experiments. DCF fluorescence intensity was quantified from the acquired images, and background-corrected DCF fluorescence intensity was calculated for each analyzed field. Background fluorescence was measured in a cell-free region of each image and subtracted from the corresponding DCF fluorescence intensity. The same background-subtraction procedure was applied consistently to all analyzed images. Measurements from the three fields within each independent biological experiment were averaged to generate one biological replicate for subsequent statistical analysis.

### 4.5 MDA assay

Malondialdehyde (MDA) levels were measured as a supportive index of lipid peroxidation-associated oxidative damage using a commercial Lipid Peroxidation MDA Assay Kit (Beyotime, Cat# S0131S). Cells were lysed using Beyotime's Western and IP Cell Lysis Buffer (Cat# P0013). Lysates were centrifuged at 10,000–12,000 g for 10 min at 4°C, and the supernatant was collected for further analysis. Protein concentration was quantified using the BCA Protein Assay Kit (Beyotime, Cat# P0010). The working reagent from the MDA kit was added to the samples in accordance with the manufacturer's instructions. After thorough mixing, the reaction mixture was incubated in a boiling water bath (100°C) for 15 min, followed by cooling to room temperature. Samples were centrifuged at 1000 g for 10 min at room temperature, and 200 μL of the resulting supernatant was transferred to a 96-well plate. The absorbance was measured at 532 nm using a microplate reader, and MDA concentration was calculated based on a standard curve.

### 4.6 Western blot analysis

Cells were lysed using RIPA buffer (Beyotime, Cat# P0013B) supplemented with protease and phosphatase inhibitors. Lysates were incubated at 4°C for 30 min and centrifuged at 12,000 g for 10 min. The supernatant was collected for subsequent analysis. Protein concentration was quantified using a BCA Protein Assay Kit. Equal amounts of protein were mixed with SDS loading buffer and denatured by boiling at 100°C for 5 min. Proteins were separated by 10–12% SDS-PAGE and transferred onto PVDF membranes (Millipore). Membranes were blocked with 5% non-fat milk or BSA at room temperature for 1 h, followed by incubation with primary antibodies overnight at 4°C. The next day, membranes were washed three times with TBST (10 min each) and then incubated with HRP-conjugated secondary antibodies for 2 h at room temperature. After washing, protein bands were visualized using enhanced chemiluminescence (ECL) reagents and imaged with a gel documentation system.

### 4.7 Ubiquitination assay

The palmitoylation-deficient GPX4-C66S mutant was designed based on previous evidence identifying Cys66 as a key ZDHHC20-targeted palmitoylation site of GPX4 [35]. For GPX4 ubiquitination analysis, HEK293T cells were co-transfected with His-tagged GPX4 or His-tagged GPX4-C66S, Myc-tagged ubiquitin, and Flag-tagged ZNF419 plasmids as indicated. Cells were treated with MG132 before harvest to inhibit proteasomal degradation. Cell lysates were prepared under denaturing or stringent lysis conditions to preserve ubiquitinated proteins. His-tagged GPX4 was immunoprecipitated using His-tag affinity beads, and ubiquitinated GPX4 was detected by immunoblotting with anti-Myc antibody. Input lysates were analyzed by Western blotting using the indicated antibodies.

### 4.8 CHX chase analysis

For CHX chase analysis, cells were treated with cycloheximide at 100 μg/mL for the indicated time points. GPX4 band intensity at each time point was first normalized to the corresponding loading control and then expressed relative to the 0-h value within the same experimental group. The degradation curves therefore represent the relative remaining GPX4 protein over time and were used to compare relative degradation kinetics independent of baseline expression differences.

### 4.9 Determination of GSH and GSSG levels

The intracellular levels of reduced glutathione (GSH) and oxidized glutathione (GSSG) were measured using a commercial assay kit (Beyotime, Cat# S0053), following the manufacturer's instructions. Cells were lysed with the lysis buffer provided, and proteins were removed from the samples using a deproteinization step as recommended in the protocol.

To specifically measure GSSG, a masking reagent was added to eliminate reduced GSH before initiating the assay. Subsequently, colorimetric detection was performed using the reagents supplied in the kit, and absorbance was measured at 412 nm using a microplate reader. GSH and GSSG concentrations were calculated based on the standard curve, and the GSH/GSSG ratio was determined to assess the cellular redox status.

### 4.10 Quantitative real-time PCR (RT-qPCR)

Total RNA was extracted from cells using the Total RNA Extraction Kit (Vazyme, Cat# R401-01) following the manufacturer's instructions. RNA concentration and purity were measured using a NanoDrop spectrophotometer. Reverse transcription was performed using HiScript® II Q RT SuperMix for qPCR (+gDNA wiper) (Vazyme, Cat# R223-01), which includes a genomic DNA removal step to synthesize cDNA.

Quantitative PCR was conducted using ChamQ Universal SYBR qPCR Master Mix (Vazyme, Cat# Q711-02) according to the manufacturer's protocol. Amplification was performed on a Roche LightCycler 480 96-well real-time PCR system. All samples were run in triplicate, and relative gene expression was calculated using the 2^-ΔΔCt method with *GAPDH* as the internal reference gene. Primer sequences used for RT-qPCR are listed below and are presented in the 5′–3′ direction.

**Table:** 

Target gene	Forward primer (5′–3′)	Reverse primer (5′–3′)
*AR*	ATGGTGAGCAGAGTGCCCTATC	ATGGTCCCTGGCAGTCTCCAAA
*ZDHHC20*	GCCTTTTCGTGGCTGCAACAGT	GCTGAAAAGTGAGAGGACGCTG
*GPX4*	ACAAGAACGGCTGCGTGGTGAA	GCCACACACTTGTGGAGCTAGA
*ZNF419*	AGGCTCCTCTACCGCAATGTGA	CACAACTTCCCAAGCAGGCATG
*SLC7A11*	TCCTGCTTTGGCTCCATGAACG	AGAGGAGTGTGCTTGCGGACAT
*GAPDH*	GTCTCCTCTGACTTCAACAGCG	ACCACCCTGTTGCTGTAGCCAA

### 4.11 ABE assay

The ABE assay was performed to detect GPX4 palmitoylation as previously described with modifications. Cells were lysed in buffer containing 50 mM Tris-HCl (pH 7.4, Beyotime, ST774), 150 mM NaCl (ST348), 5 mM EDTA (ST063), and 2.5% SDS (ST629), supplemented with protease and phosphatase inhibitors. Lysates were sonicated, centrifuged at 13,000 g for 5 min at 4°C, and quantified by BCA assay. For each sample, 200 μg protein was reduced with 10 mM TCEP (ST049) at room temperature for 30 min and alkylated with 25 mM N-ethylmaleimide (NEM, ST3321) for 2 h in the dark. Proteins were precipitated using CHCl_3_/MeOH/H_2_O (1:4:3), washed twice with 80% methanol, and air-dried.

Pellets were resuspended in lysis buffer containing 3 mM biotin-HPDP (Y170622) and incubated with either 1 M hydroxylamine (HAM, ST2434, pH 7.0) or 1 M NaCl as negative control for 3 h at room temperature. Proteins were re-precipitated and resuspended in suspension buffer (50 mM Tris-HCl, 5 mM EDTA, 2% SDS, 8 M urea [ST2027], plus inhibitors). Biotinylated proteins were enriched using 20 μL streptavidin magnetic beads (P2151) by overnight incubation at 4°C with rotation, followed by three washes with 1% SDS in PBS. Bound proteins were eluted with 2× SDS loading buffer at 100°C for 15 min and analyzed by Western blot with anti-GPX4 antibody.

### 4.12 ChIP-qPCR

ChIP-qPCR assays were performed using the Pierce™ Magnetic ChIP Kit (Thermo Fisher Scientific, Cat# 26157) according to the manufacturer's instructions. AR ChIP-qPCR assays targeting the predicted ARE-containing regions of the ZNF419 promoter were performed in AR-positive LNCaP and 22Rv1 prostate cancer cells. ZNF419 ChIP-qPCR assays targeting the candidate ZDHHC20 enhancer region were performed in 22Rv1 and LNCaP cells. H3K27ac ChIP-qPCR assays targeting the same candidate enhancer region were also performed in 22Rv1 and LNCaP cells under the indicated ZNF419 expression conditions.

Briefly, cells were crosslinked with 1% formaldehyde for 10 min at room temperature, and crosslinking was quenched with 125 mM glycine for 5 min. Cells were lysed, and chromatin was enzymatically digested followed by mild sonication to obtain DNA fragments ranging from 150 to 900 bp. The lysates were incubated overnight at 4°C with the indicated specific antibodies or normal IgG as a negative control. Immune complexes were captured with Protein A/G magnetic beads and washed extensively. Crosslinks were reversed, and DNA was purified using spin columns.

Purified DNA was analyzed by quantitative real-time PCR using ChamQ Universal SYBR qPCR Master Mix on a QuantStudio™ 6 Flex Real-Time PCR System (Thermo Fisher Scientific). Primers targeting the predicted ARE-containing regions of the ZNF419 promoter or the candidate ZDHHC20 enhancer region were used as indicated. Data were normalized as indicated and presented as either % input or fold enrichment relative to the corresponding IgG control. The PSA/KLK3 locus was included as a positive-control region for AR ChIP-qPCR.

**Table:** 

Target region	Forward primer (5′–3′)	Reverse primer (5′–3′)
ARsite1	GAGAAGCTGGTTGTGCGCT	GCTGGAGGGGAGACAATGAC
ARsite2	GCTGCCATGGAAGGGGTAAA	ATGAAGCCCTCCCTCCTTCT
ARsite3	CTACCCACCTGTGAGGAACTG	AATAGGCGGCAAACCCATGA
ZD20-ChIP	GACGTGACCTCCACCCCTAG	TGCCTGCGATCTGACTCTGC
PSA-CHIP	GTGTGTGGACCTCCATGTTATT	CCACTCACCTTTCCCCTCAAG

### 4.13 Dual-luciferase® reporter assay

Dual-Luciferase® Reporter Assay System (Promega, Cat# E1910) was used to measure firefly and Renilla luciferase activities according to the manufacturer's protocol. For *ZNF419* promoter reporter assays, 293T cells were seeded in 24-well plates and co-transfected with wild-type or ARE-deleted *ZNF419* promoter firefly luciferase reporter constructs, Renilla luciferase control plasmid, and AR expression plasmid where indicated. Transfections were performed using Lipofectamine 3000 according to the manufacturer's instructions.

After 24–48 h, cells were lysed with Passive Lysis Buffer. Firefly luciferase activity was measured using Luciferase Assay Reagent II, followed by Renilla luciferase measurement using Stop & Glo® Reagent. Firefly luciferase activity was normalized to Renilla luciferase activity to control for transfection efficiency.

### 4.14 Establishment of ZNF419 knockdown or overexpression cell lines

To stably knock down *ZNF419* expression, the pLV3-U6-ZNF419(human)-shRNA-Puro plasmid was constructed, with shRNA target sequences of 5′-GCAATGTGATGCTGGAGAA-3′ (#2) and 5′-GCAGCACTGTGGAGAGAAA-3′ (#3). ZNF419 overexpression plasmids, shRNA vectors, and ZDHHC20 overexpression plasmids were purchased from MiaoLing Bio. Lentiviruses were packaged in 293T cells and used to infect target cells. Stable cell lines were selected using 1 μg/ml puromycin (Beyotime, Cat. No. ST551).

**Table:** 

Targeting construct	Target sequence (5′–3′)
shZNF419(#1)	5′-GCTTACAGACCATGAGGAGCA-3′
shZNF419(#2)	5′-GCAATGTGATGCTGGAGAA-3′
shZNF419(#3)	5′-GCAGCACTGTGGAGAGAAA-3′
shAR(#1)	5′-CACCAATGTCAACTCCAGGAT-3′
shAR(#2)	5′-GAGCGTGGACTTTCCGGAAAT-3′
Enhancer sg1	5′-GTCGGGCAGAGTCAGATCGC-3′

### 4.15 CRISPR/Cas9-mediated enhancer perturbation

CRISPR/Cas9-mediated perturbation of the candidate *ZDHHC20* enhancer region was performed in 293T cells because of their high transfection and genome-editing efficiency. Cells were transfected with plasmids expressing Cas9 and either control sgRNA or enhancer-targeting sgRNA. After transfection, cells were harvested for RNA extraction and RT-qPCR analysis of *ZDHHC20* expression.

To assess sgRNA-mediated perturbation of the intended enhancer locus, genomic DNA was extracted from cells transfected with control sgRNA or enhancer-targeting sgRNA. The targeted enhancer region was analyzed by PCR/qPCR using primers located within or adjacent to the intended editing region, and the relative genomic DNA signal was normalized to the control group.

### 4.16 Immunofluorescence

Cells grown on coverslips were fixed in 4% paraformaldehyde, permeabilized with 0.1% Triton X-100, and blocked with 5% BSA. After incubation with primary and fluorescent secondary antibodies, nuclei were counterstained with DAPI. Images were captured using a laser scanning confocal microscope (Leica SP8).

### 4.17 Immunohistochemistry (IHC)

Formalin-fixed, paraffin-embedded tissue sections were cut into 4-μm-thick sections, deparaffinized in xylene, and rehydrated through a graded ethanol series. Antigen retrieval was performed using citrate antigen retrieval buffer (MXB Biotechnologies, MVS-0103, pH 6.0) under high-temperature and high-pressure conditions. Specifically, sections for ZNF419 staining were retrieved for 8 min, whereas sections for ZDHHC20, GPX4, 4-HNE, and Ki-67 staining were retrieved for 5 min. After blocking endogenous peroxidase activity, sections were incubated with the corresponding primary antibodies, including anti-ZNF419 antibody at 1:100 dilution, anti-ZDHHC20 antibody at 1:200 dilution, anti-GPX4 antibody at 1:200 dilution, anti-4-HNE antibody at 1:200 dilution, and ready-to-use anti-Ki-67 antibody. Immunostaining was performed using the UltraSensitive™ SP IHC Kit (Mouse/Rabbit) (MXB Biotechnologies, KIT-9720) according to the manufacturer's instructions, followed by DAB chromogenic development and hematoxylin counterstaining. The sections were then dehydrated, cleared, and mounted. Negative controls were prepared by omitting the primary antibody.

Staining intensity and the proportion of positive cells were independently evaluated by two pathologists who were blinded to the clinical information and experimental group allocation, where applicable. Each pathologist independently assigned an H-score ranging from 0 to 300 for each marker in each tissue specimen. The mean of the two independently assigned H-scores was calculated for each marker and specimen and used as the continuous value for statistical analysis. The mean H-score was also converted into one of four predefined ordinal staining categories: 0, negative or minimal staining (H-score 0–10); 1, weak staining (11–100); 2, moderate staining (101–200); and 3, strong staining (201–300). Both the continuous mean H-scores and the H-score-derived ordinal categories were analyzed.

Inter-observer agreement was evaluated separately for each marker in tumor and adjacent non-tumor tissues (n = 30 specimens per tissue type) using a two-way mixed-effects, absolute-agreement intraclass correlation model. Single-rating ICC(A,1) and average-rating ICC(A,2), the latter corresponding to the mean of the two pathologists' ratings, were calculated with approximate 95% confidence intervals using Pingouin version 0.5.5. Categorical concordance was assessed by applying the predefined H-score thresholds separately to each pathologist's independently assigned score. For specimens with category-level differences between the two pathologists, the final continuous H-score was defined as the mean of the two independently assigned scores, and the final ordinal category was derived from this mean.

### 4.18 Cell viability assay (CCK-8)

Cell proliferation was measured using a Cell Counting Kit-8 (CCK-8; Beyotime, C0039) according to the manufacturer's protocol. Absorbance at 450 nm was recorded using a microplate reader. All experiments were performed in triplicate.

For RSL3 dose–response rescue assays, NC, shZNF419-2, and shZNF419-3 cells were treated with RSL3 at 0, 0.5, 1, 2, or 4 μM in the absence or presence of Fer-1 (10 μM) for 24 h. Cell viability was measured using the CCK-8 assay in three independent biological experiments. Dose–response curves were fitted using nonlinear regression with a variable-slope model. IC50 estimates were interpreted as experimentally bounded only when 50% viability was reached within the tested concentration range. For the Fer-1-containing groups, in which 50% viability was not reached at concentrations up to 4 μM RSL3, fitted IC50 estimates were treated as model-based extrapolations.

### 4.19 Transwell migration assay

Cell migration was assessed using Transwell chambers with 8 μm pore size membranes (Corning). Cells were seeded in the upper chamber in serum-free medium, while the lower chamber contained medium supplemented with 10% FBS as a chemoattractant. After incubation, migrated cells on the lower surface were fixed with paraformaldehyde, stained with crystal violet, and counted under a microscope.

### 4.20 Xenograft model in nude mice

All animal experiments were approved by the Animal Ethics Committee of China Medical University. Four-week-old male BALB/c nude mice were maintained under specific pathogen-free conditions with free access to food and water.

For xenograft establishment, 22Rv1 cells with stable transduction were subcutaneously injected into the axillary region of each mouse. For the RSL3 xenograft experiment (Experiment 1), tumor size was measured every two days using digital calipers. Tumor volume was calculated using the following formula: volume = a × b^2^ × 0.52, where a represents the longest diameter and b represents the shortest diameter. When tumors reached approximately 50–60 mm^3^, mice were randomly assigned to the indicated treatment groups. Unless otherwise indicated, each group contained n = 5 mice. No formal a priori power calculation was performed; the group size of five mice was selected based on prior xenograft experience and experimental feasibility. Tumor measurements were performed by investigators blinded to group allocation whenever possible. Immunohistochemical evaluation was performed by investigators/pathologists blinded to the treatment groups.

#### 4.20.1 Experiment 1

To explore whether ZNF419 depletion alters tumor responses under experimental GPX4-proximal pharmacological challenge, mice bearing NC, shZNF419-2, or shZNF419-3 xenografts were further divided into vehicle-treated or RSL3-treated groups. RSL3 was administered at 100 mg/kg by intraperitoneal injection every three days for 12 days. The vehicle solution consisted of 5% DMSO, 40% PEG300, 5% Tween 80, and 50% ddH_2_O. Vehicle-treated mice received the corresponding vehicle solution according to the same schedule.

#### 4.20.2 Experiment 2

To evaluate tumor responses to IKE in the context of ZNF419 depletion and enzalutamide treatment, mice were randomly assigned to six groups (n = 5 mice per group): NC + vehicle, NC + IKE, shZNF419-3 + vehicle, shZNF419-3 + IKE, NC + enzalutamide, and NC + enzalutamide + IKE. IKE was administered intraperitoneally at 40 mg/kg every 4 days over a 20-day treatment period. IKE was formulated in a vehicle containing 10% DMSO, 40% PEG300, 5% Tween 80, and 45% sterile normal saline. The IKE working solution was prepared at 4 mg/mL, corresponding to an injection volume of 10 mL/kg. Enzalutamide was administered at 10 mg/kg/day by oral gavage throughout the same 20-day treatment period. Mice in the combination group received both agents according to their respective dosing schedules. Vehicle-treated mice received the corresponding drug-free vehicle formulations through the same administration routes and according to the matched dosing schedules. Body weights were recorded on days 0, 4, 8, 12, 16, and 20 throughout the treatment period as a general indicator of treatment tolerability.

#### 4.20.3 Supplementary exploratory experiment

In the earlier enzalutamide-focused xenograft experiment, mice were assigned to four groups: NC + vehicle, NC + enzalutamide, shZNF419-3 + enzalutamide, and NC + enzalutamide + RSL3. Enzalutamide was administered at 10 mg/kg/day by oral gavage throughout the 20-day treatment period. RSL3 was administered intraperitoneally at 100 mg/kg every 3 days over the same treatment period. Mice in the combination group received both agents according to their respective dosing schedules. Because this experiment did not constitute a complete factorial design and systemic RSL3 administration has pharmacokinetic and interpretive limitations, the results are presented as exploratory supplementary evidence.

At the experimental endpoint, mice were euthanized, and tumors were harvested for tumor weight measurement, Western blot analysis, and histological analyses.

### 4.21 Bioinformatic analysis of public transcriptomic datasets

Publicly available transcriptomic datasets GSE248895, GSE164880, and GSE232034 were obtained from the Gene Expression Omnibus database. GSE248895 was used to represent AR perturbation, GSE164880 was used to compare enzalutamide-resistant and parental prostate cancer cells, and GSE232034 was used to examine erastin-treated prostate cancer cells. Because GSE232034 contained separate comparisons involving LNCaP and PC3 cells, these comparisons were analyzed separately, resulting in four gene lists: GSE248895, GSE164880, GSE232034-LNCaP, and GSE232034-PC3.

Differential-expression analysis for the relevant comparisons was performed using GEO2R. Genes with a nominal P value < 0.05 and |log2 fold change| > 0.4 were retained. The filtered gene lists were exported from GEO2R and imported into R. Duplicate gene entries were removed before intersection analysis. A four-set Venn diagram was generated using the VennDiagram package. To further visualize the size and structure of the intersections, a binary gene-membership matrix was constructed, in which each gene was coded as present or absent in GSE248895, GSE164880, GSE232034-LNCaP, and GSE232034-PC3, and an UpSet plot was generated using the UpSetR package. Genes present in all four filtered gene sets were identified by requiring membership in all four datasets. Both upregulated and downregulated genes meeting the predefined thresholds were included, and concordant directionality across the four comparisons was not required. The intersection established a shared candidate pool and was not used to rank the six genes by effect size or statistical significance.

### 4.22 Statistical analysis

Statistical analyses were performed using GraphPad Prism 9.0 (GraphPad Software) and Python, with Pingouin version 0.5.5 used for inter-observer reliability analyses. Unless otherwise indicated, quantitative data are presented as mean ± SD from three independent biological experiments. Technical replicates, when included, were averaged within each biological replicate before statistical analysis. Statistical tests were performed using independent biological replicates as the unit of analysis.

Endpoint tumor weights and endpoint tumor volumes in the six-group IKE xenograft experiment were analyzed separately using ordinary one-way ANOVA followed by Tukey's multiple-comparisons test. For each endpoint, all 15 pairwise comparisons among the six experimental groups were evaluated within a single comparison family, with the family-wise error rate controlled at 0.05. Individual mice were the experimental units (n = 5 per group). The individual pairwise contrasts were not prospectively pre-specified before data analysis and were therefore interpreted as post hoc exploratory comparisons.

For comparisons between two groups, a two-tailed Student's t-test was used for normally distributed quantitative data. For comparisons among multiple groups, one-way ANOVA followed by Tukey's post hoc test was used. Tumor growth curves were analyzed using two-way repeated-measures ANOVA, with time as the repeated-measures factor and treatment group as the between-subject factor. Kaplan–Meier survival curves were compared using the log-rank test. For the clinical IHC cohort, the mean of the two pathologist-assigned H-scores was analyzed as a continuous measure and was also converted into the predefined ordinal staining categories. Paired tumor and adjacent non-tumor values were compared using two-sided Wilcoxon matched-pairs signed-rank tests. Pairwise relationships among ZNF419, ZDHHC20, and GPX4 and monotonic associations with ISUP grade group or Gleason score were assessed using two-sided Spearman rank correlation analysis. Differences in continuous mean H-scores across ISUP grade groups were evaluated using the Kruskal–Wallis test. Benjamini–Hochberg correction was applied separately to the three pairwise protein correlations and the three marker-wise Kruskal–Wallis comparisons; nominal and adjusted p values are reported where applicable. A value of p < 0.05 was considered statistically significant after multiple-comparison correction where applied.

## CRediT authorship contribution statement

**Yuanxiang Hou:** Writing – review & editing, Writing – original draft, Visualization, Validation, Formal analysis, Conceptualization. **Wenyan Zhao:** Visualization, Validation, Data curation. **Jingyi Liu:** Validation. **Ruiping Tang:** Validation. **Tong Deng:** Validation. **Huaiyu Zhu:** Investigation. **Hiroshi Miyamoto:** Writing – review & editing. **Yuyan Zhu:** Writing – review & editing, Writing – original draft, Supervision. **Ming Sun:** Writing – review & editing, Writing – original draft, Visualization, Resources, Funding acquisition. **Guiyang Jiang:** Writing – review & editing, Writing – original draft, Visualization, Supervision, Funding acquisition, Conceptualization.

## Ethics declaration

Written informed consent to take part in the study and to publish the article has been obtained from all participants or their legal representatives. The privacy rights of participants have been observed.

This study included organ or tissue donors. This study includes human biological material and consent was obtained by donors, or their next of kin or legal representatives, for use in this study and for publication of the article. The samples used in this research were not sourced from executed prisoners or prisoners of conscience.

This study was performed in compliance with relevant laws, regulatory frameworks and guidelines where the research took place. This study was conducted in accordance with Declaration of Helsinki. This study was approved by the Medical Scientific Research Ethics Committee, The First Hospital of China Medical University. (Approval No. Kelunshen [2026] No. 30)

This study was conducted in accordance with the ARRIVE (Animal Research: Reporting of In Vivo Experiments) guidelines. This study was approved by the Laboratory Animal Welfare Ethics Committee of China Medical University. (Approval No. CMU20260823)

## Significance statement

AR-regulated ZNF419 is associated with reduced ferroptosis susceptibility in prostate cancer cells, at least in part through a ZDHHC20-associated regulatory mechanism that contributes to palmitoylation-associated GPX4 stabilization, revealing a mechanistic link between androgen signaling and redox regulation.

## Declaration of generative AI and AI-assisted technologies in the manuscript preparation process

During the preparation of this work, the authors used ChatGPT (OpenAI) for language editing and assistance in refining manuscript wording. After using this tool, the authors reviewed and edited the content as needed and take full responsibility for the content of the published article.

## Declaration of competing interest

The authors declare that they have no known competing financial interests or personal relationships that could have appeared to influence the work reported in this paper.

## Data Availability

The data analyzed in this study were obtained from publicly available repositories. Specifically, the transcriptomic datasets GSE248895 (AR perturbation), GSE232034 (erastin-induced ferroptosis), and GSE164880 (enzalutamide-resistant versus parental prostate cancer cell lines) were downloaded from the Gene Expression Omnibus (GEO). Publicly available ChIP-seq data used in this study included AR ChIP-seq data from GEO sample accession GSM980647 and ZNF419 ChIP-seq data from GEO sample accessions GSM2466550 and GSM2466549. Additional public resources, including UALCAN, TIMER2.0, GEPIA2, Cistrome DB, UCSC Genome Browser, and SCREEN, were used as described in the relevant sections. All other data generated in this study are available within the article and its Supplementary Data files. Uncropped Western blot images with molecular-weight markers are provided in the accompanying Supplementary Source Data file.
