# Supplementary material for: Androgen receptor-regulated ZNF419 modulates ferroptosis sensitivity via the ZDHHC20–GPX4 pathway in prostate cancer
Source: Redox Biol. 2026 Oct 8;97:104433. doi: 10.1016/j.redox.2026.104433 (PMC13678104; doi:10.1016/j.redox.2026.104433)
Supplement: Supplementary file 1 [file mmc1.docx]

**Supplementary Figure 1. ZNF419 expression and knockdown validation in prostate cancer cells.**

(A) Expression of ZNF419 across tumor types from the UALCAN database.

(B) ZNF419 expression in primary prostate cancer and normal prostate tissues from the UALCAN database.

(C) Western blot analysis and quantification of AR and ZNF419 protein expression in prostate cancer cell lines. Protein levels were normalized to GAPDH.

(D) Western blot analysis of ZNF419 knockdown efficiency using three independent shRNA sequences in 22Rv1 cells.

(E, F) Quantification of ZNF419 knockdown efficiency in 22Rv1 (E) and LNCaP (F) cells. Protein levels were normalized to GAPDH.

(G) Transwell migration assay showing reduced migration capacity after ZNF419 knockdown in 22Rv1 and LNCaP cells.

Data are presented as mean ± SD from three independent biological experiments. Quantification of ZNF419 knockdown in panels E and F and migration data in panel G were analyzed using one-way ANOVA followed by Tukey’s multiple-comparisons test. *p < 0.05, **p < 0.01, ***p < 0.001, ****p < 0.0001; ns, not significant.

**Supplementary Figure 2. AR signaling regulates ZNF419 expression in AR-positive prostate cancer cells.**

(A, B) Heatmaps showing the relative protein expression levels of AR and ZNF419 in 22Rv1 (A) and LNCaP (B) cells after treatment with increasing concentrations of DHT. Values represent the average of three independent biological replicates.

(C, D) Heatmaps showing the relative protein expression levels of AR and ZNF419 in AR-negative PC3 (C) and DU145 (D) cells after treatment with increasing concentrations of DHT. Values represent the average of three independent biological replicates.

(E, F) Quantification of AR and ZNF419 protein expression in 22Rv1 (E) and LNCaP (F) cells following AR knockdown with two independent shRNA sequences. Protein levels were normalized to GAPDH.

(G–I) UCSC Genome Browser tracks showing publicly available AR ChIP-seq signals across the genomic region surrounding the ZNF419 locus. The AR ChIP-seq track was derived from the GEO sample accession GSM980647. The red box indicates a promoter-proximal AR enrichment signal near the ZNF419 transcription start site (TSS), while additional distal AR peaks were also observed within the broader genomic neighborhood. Genome build: hg38.

Data are presented as mean ± SD from three independent biological experiments where quantitative data are shown. Heatmaps in panels A–D show mean relative protein expression values from three independent biological experiments. Quantitative data in panels E and F were analyzed using two-way ANOVA followed by Tukey’s multiple-comparisons test. *p < 0.05, **p < 0.01, ***p < 0.001, ****p < 0.0001; ns, not significant.

**Supplementary Figure 3. Additional analyses of RSL3 sensitivity, GPX4 protein stability, and ferroptosis-associated markers in ZNF419-manipulated prostate cancer cells and xenografts.**

(A, B) RSL3 dose–response rescue assays in NC, shZNF419-2, and shZNF419-3 22Rv1 (A) and LNCaP (B) cells treated with RSL3 at 0, 0.5, 1, 2, or 4 μM in the absence or presence of Fer-1 (10 μM) for 24 h. IC50 values were calculated using nonlinear regression with a variable-slope model. For Fer-1-containing groups in which 50% viability was not reached within the tested RSL3 concentration range (0–4 μM), fitted IC50 values were regarded as model-based extrapolations rather than experimentally bounded estimates.

(C, D) Heatmaps showing relative protein expression of ferroptosis-associated markers in ZNF419-depleted 22Rv1 (C) and LNCaP (D) cells.

(E, F) CHX-chase analysis of GPX4 protein stability following ZNF419 overexpression in 22Rv1 (E) and LNCaP (F) cells. Cells were treated with cycloheximide (CHX; 100 μg/mL) for 0, 12, 24, 36, or 48 h before harvest. Heatmaps and corresponding quantification curves are shown.

(G, H) CHX-chase analysis of GPX4 protein stability following ZNF419 knockdown in 22Rv1 (G) and LNCaP (H) cells. Cells were treated with CHX (100 μg/mL) for 0, 6, or 12 h before harvest. Heatmaps and corresponding quantification curves are shown.

(I, J) Heatmaps showing GPX4 protein expression in ZNF419-depleted LNCaP (I) and 22Rv1 (J) cells treated with or without MG132 (10 μM) for 8 h.

(K) Heatmaps showing relative ZNF419 and GPX4 protein expression in xenograft tumor tissues from the indicated groups treated with vehicle or RSL3. RSL3 was administered intraperitoneally at 100 mg/kg every 3 days for 12 days, and vehicle-treated mice received the corresponding vehicle according to the same schedule.

(L) Representative IHC images of ZNF419, GPX4, and 4-HNE staining in xenograft tumors from the indicated groups treated with vehicle or RSL3 (100 mg/kg, intraperitoneally, every 3 days for 12 days).

Data are presented as mean ± SD from three independent biological experiments where applicable. Dose–response curves in panels A and B were fitted using nonlinear regression to estimate IC50 values. CHX-chase data in panels E–H were normalized to the corresponding loading control and expressed relative to the 0-h value within each group. Heatmaps in panels C, D, and I–K show mean relative protein expression values.

**Supplementary Figure 4. Additional validation of the ZNF419–ZDHHC20–GPX4 regulatory pathway and AR-independent ZNF419 regulation.**

(A, B) Heatmaps showing GPX4 protein expression in ZNF419-overexpressing 22Rv1 (A) and LNCaP (B) cells treated with or without 2-BP (10 μM) for 24 h. Values represent the mean relative protein expression from three independent biological experiments.

(C) Correlation analysis between ZNF419 and ZDHHC20 expression using the GEPIA2 database.

(D, E) Heatmaps showing ZNF419, ZDHHC20, and GPX4 protein expression in 22Rv1 (D) and LNCaP (E) cells following ZNF419 knockdown. Values represent the average of three independent biological replicates.

(F) Relative genomic DNA signal at the targeted candidate ZDHHC20 enhancer region in HEK293T cells transfected with control sgRNA or enhancer-targeting sgRNA. Genomic DNA PCR/qPCR analysis was performed to assess sgRNA-mediated perturbation of the intended enhancer locus.

(G, H) Representative fluorescence microscopy images of DCFH-DA staining showing ROS accumulation in 22Rv1 (G) and LNCaP (H) cells following RSL3 treatment, with or without Fer-1 rescue. The indicated groups include control cells, ZNF419-knockdown cells, and the corresponding groups with ZDHHC20 overexpression.

(I, J) Quantification of corrected DCF fluorescence intensity following erastin treatment, with or without Fer-1 rescue, in 22Rv1 (I) and LNCaP (J) cells. These quantitative data correspond to the representative fluorescence images shown in Fig. 6P and Q.

(K, L) Quantification of corrected DCF fluorescence intensity following RSL3 treatment, with or without Fer-1 rescue, in 22Rv1 (K) and LNCaP (L) cells. These quantitative data correspond to the representative fluorescence images shown in panels G and H.

(M, N) Western blot analysis of AR and ZNF419 protein expression in 22Rv1 (M) and LNCaP (N) cells following short-term stimulation with DHT (5 nM) for 0, 10, 20, or 30 min, or 1, 2, 3, or 6 h.

(O, P) Western blot analysis of AR, ERK1/2 phosphorylation, and ZNF419 expression in 22Rv1 (O) and LNCaP (P) cells following DHT stimulation (5 nM, 6 h) with or without U0126 (5 or 15 μM).

(Q, R) Western blot analysis of AR, phosphorylated ERK1/2, total ERK1/2, and ZNF419 expression in AR-negative PC3 (Q) and DU145 (R) cells following 6 h treatment with U0126 (5 or 15 μM).

(S, T) RT-qPCR analysis of ZNF419 and SLC7A11 mRNA expression following ZNF419 knockdown in 22Rv1 (S) and LNCaP (T) cells.

(U, V) Western blot analysis of GPX4 protein expression in AR-negative PC3 (U) and DU145 (V) cells following ZNF419 knockdown.

Data are presented as mean ± SD from three independent biological experiments where quantitative data are shown. Heatmaps in panels A, B, D, and E show mean relative protein expression values obtained from three independent biological experiments. Correlation analysis in panel C was performed using Pearson’s correlation test. ROS fluorescence quantification in panels I–L and RT-qPCR data in panels S and T were analyzed using two-way ANOVA followed by Tukey’s multiple-comparisons test. Panels G and H show representative fluorescence microscopy images from three independent biological experiments. *p < 0.05, **p < 0.01, ***p < 0.001, ****p < 0.0001; ns, not significant.

**Supplementary Figure 5. Enzalutamide-focused xenograft experiment using systemic RSL3 as an exploratory GPX4-proximal pharmacological challenge.**

(A, B) Representative images of tumor-bearing mice (A) and excised xenograft tumors (B) from the NC + vehicle, NC + ENZ, shZNF419-3 + ENZ, and NC + ENZ + RSL3 groups. ENZ was administered at 10 mg/kg/day by oral gavage throughout the 20-day treatment period, whereas RSL3 was administered intraperitoneally at 100 mg/kg every 3 days over the same treatment period.

(C) Tumor weight analysis of xenografts from the indicated groups.

(D) Tumor volume analysis of xenografts from the indicated groups.

(E) Representative IHC staining of ZNF419, ZDHHC20, GPX4, 4-HNE, and Ki-67 in xenograft tumor tissues. Negative-control staining was performed by omission of the primary antibody. Scale bars are indicated in the images.

(F) Western blot analysis of ZNF419, ZDHHC20, and GPX4 protein expression in xenograft tumor tissues. GAPDH was used as the loading control.

(G) Quantification of Western blot results shown in panel F.

Data are presented as mean ± SD. Xenograft experiments included five mice per group. Tumor weights and endpoint tumor volumes were analyzed using one-way ANOVA followed by Tukey’s multiple-comparisons test. Western blot quantification in panel G was analyzed separately for each protein using ordinary one-way ANOVA followed by Tukey’s multiple-comparisons test. This experiment is presented as exploratory supplementary evidence because of the pharmacokinetic and interpretive limitations of systemic RSL3 administration. *p < 0.05, **p < 0.01, ***p < 0.001, ****p < 0.0001.

**Supplementary Fig. 6. Pairwise associations among ZNF419, ZDHHC20, and GPX4 expression in human prostate cancer tissues.**

Scatter plots show continuous mean H-scores for 30 prostate cancer specimens. Each point represents one patient. Pairwise associations were assessed using two-sided Spearman rank correlation analysis. Benjamini–Hochberg-adjusted P values were calculated across the three pairwise marker correlations. (A) ZNF419 versus ZDHHC20. (B) ZNF419 versus GPX4. (C) ZDHHC20 versus GPX4.
