# Supplementary material for: Androgen receptor-regulated ZNF419 modulates ferroptosis sensitivity via the ZDHHC20–GPX4 pathway in prostate cancer
Source: Redox Biol. 2026 Oct 8;97:104433. doi: 10.1016/j.redox.2026.104433 (PMC13678104; doi:10.1016/j.redox.2026.104433)
Supplement: Supplementary file 2 [file mmc2.docx]

Figure 1H

AR

AR+White

ZNF419

ZNF419+White

GAPDH

GAPDH+White

White

180

130

100

70

55

40

35

25

15

Figure 1J

ZNF419

ZNF419+White

GAPDH

GAPDH+White

White

70

55

40

35

25

Figure 1K

ZNF419

ZNF419+White

GAPDH

GAPDH+White

White

180

130

100

70

55

40

35

25

Figure 2B

AR

AR+White

ZNF419

ZNF419+White

GAPDH

GAPDH+White

White

180

130

100

70

55

40

35

25

Figure 2C

AR

AR+White

ZNF419

ZNF419+White

GAPDH

GAPDH+White

White

180

130

100

70

55

40

35

25

Figure 2D

AR

AR+White

ZNF419

ZNF419+White

GAPDH

GAPDH+White

White

180

130

100

70

55

40

35

25

15

Figure 2E

AR

AR+White

ZNF419

ZNF419+White

GAPDH

GAPDH+White

White

180

130

100

70

55

40

35

25

15

Figure 2F

AR

AR+White

ZNF419

ZNF419+White

GAPDH

GAPDH+White

White

180

130

100

70

55

40

35

25

15

Figure 2G

AR

AR+White

ZNF419

ZNF419+White

GAPDH

GAPDH+White

White

180

130

100

70

55

40

35

25

Figure 4A

SLC7A11

SLC7A11+White

ACSL4

ACSL4+White

ZNF419

ZNF419+White

GAPDH

GAPDH+White

White1

180

130

100

70

55

40

35

25

15

White2

180

130

100

70

55

40

35

25

15

GPX4

GPX4+White

ZNF419

ZNF419+White

GAPDH

GAPDH+White

White 3

180

130

100

70

55

40

35

25

15

DHODH

DHODH+White

FSP1

FSP1+White

ZNF419

ZNF419+White

GAPDH

GAPDH+White

White 4

180

130

100

70

55

40

35

25

Figure 4B

SLC7A11

SLC7A11+White

ACSL4

ACSL4+White

ZNF419

ZNF419+White

GAPDH

GAPDH+White

White 1

180

130

100

70

55

40

35

25

15

DHODH

DHODH+White

FSP1

FSP1+White

GAPDH

GAPDH+White

ZNF419

ZNF419+White

White 2

180

130

100

70

55

40

35

25

15

GPX4

GPX4+White

ZNF419

ZNF419+White

GAPDH

GAPDH+White

White

180

130

100

70

55

40

35

25

15

Figure 4E

Flag-ZNF419

Flag-ZNF419+White

GAPDH

GAPDH+White

GPX4

GPX4+White

White

180

130

100

70

55

40

35

25

15

Figure 4F

Flag-ZNF419

Flag-ZNF419+White

GPX4

GPX4+White

GAPDH

GAPDH+White

White

180

130

100

70

55

40

35

25

15

Figure 4G

ZNF419

ZNF419+White

GPX4

GPX4+White

GAPDH

GAPDH+White

White

180

130

100

70

55

40

35

25

15

Figure 4H

ZNF419

ZNF419+White

GPX4

GPX4+White

GAPDH

GAPDH+White

White

180

130

100

70

55

40

35

25

15

Figure 4I

ZNF419

ZNF419+White

GPX4

GPX4+White

White

180

130

100

70

55

40

35

25

15

Figure 4J

ZNF419

ZNF419+White

GPX4

GPX4+White

GAPDH

GAPDH+White

White

180

130

100

70

55

40

35

25

15

Figure 4K

Myc-UB

Myc-UB+White

180

130

100

70

55

40

35

25

15

His-GPX4

His-GPX4+White

Input：Flag-ZNF419

Input：Flag-ZNF419+White

Input：GAPDH

Input：GAPDH+White

Input：HisGPX4

Input：GPX4+White

White

180

130

100

70

55

40

35

25

15

Figure 4P

DMSO

ZNF419

ZNF419+White

GAPDH

GAPDH+White

GPX4

GPX4+White

White

180

130

100

70

55

40

35

25

15

RSL3

ZNF419

ZNF419+White

GAPDH

GAPDH+White

GPX4

GPX4+White

White

180

130

100

70

55

40

35

25

15

Figure 5C

Flag-ZNF419

Flag-ZNF419+White

GAPDH

GAPDH+White

GPX4

GPX4+White

White

180

130

100

70

55

40

35

25

15

Figure 5D

Flag-ZNF419

Flag-ZNF419+White

GAPDH

GAPDH+White

GPX4

GPX4+White

White

180

130

100

70

55

40

35

25

15

Figure 5E

ZDHHC20

ZDHHC20+White

180

130

100

70

55

40

35

25

15

ZNF419

ZNF419+White

GAPDH

GAPDH+White

GPX4

GPX4+White

Figure 5F

ZNF419

ZNF419+White

ZDHHC20

ZDHHC20+White

GPX4

GPX4+White

GAPDH

GAPDH+White

White

180

130

100

70

55

40

35

25

15

Figure 6A

Input：ZNF419

Input：ZNF419+White

Input：GPX4

Input：GPX4+White

Input：GAPDH

Input：GAPDH+White

Input：White

180

130

100

70

55

40

35

25

15

Palm-GPX4

Palm-GPX4+White

180

130

100

70

55

40

35

25

15

Figure 6B

Input：ZNF419

Input：ZNF419+White

Input：GPX4

Input：GPX4+White

Input：GAPDH

Input：GAPDH+White

Input：White

180

130

100

70

55

40

35

25

15

Palm-GPX4

Palm-GPX4+White

180

130

100

70

55

40

35

25

Figure 6C

Flag-ZNF419

Flag-ZNF419+White

GPX4

GPX4+White

GAPDH

GAPDH+White

White

180

130

100

70

55

40

35

25

Myc-UB

Myc-UB+White

180

130

100

70

55

40

35

25

His-GPX4

His-GPX4+White

180

130

100

70

55

40

35

25

Figure 7F

ZNF419

ZNF419+White

ZDHHC20

ZDHHC20+White

GPX4

GPX4+White

GAPDH

GAPDH+White

White

180

130

100

70

55

40

35

25

15

Supplementary Figure 4M

AR

ZNF419

GAPDH

White

180

130

100

70

55

40

35

25

Supplementary Figure 4N

AR

ZNF419

GAPDH

White

180

130

100

70

55

40

35

25

Supplementary Figure 4O

AR

AR+White

P44/42

P44/42+White

P-P44/42

P-P44/42+White

ZNF419

ZNF419+White

GAPDH

White

180

130

100

70

55

40

35

25

15

Supplementary Figure 4P

AR

AR+White

P44/42

P44/42+White

P-P44/42

P-P44/42+White

ZNF419

ZNF419+White

GAPDH

GAPDH+White

White

180

130

100

70

55

40

35

25

Supplementary Figure 4Q

AR

AR+White

P44/42

P44/42+White

P-P44/42

P-P44/42+White

ZNF419

ZNF419+White

GAPDH

GAPDH+White

Supplementary Figure 4R

AR

AR+White

P44/42

P44/42+White

P-P44/42

P-P44/42+White

ZNF419

ZNF419+White

GAPDH

GAPDH+White

Supplementary Figure 4U

ZNF419

ZNF419+White

GPX4

GPX4+White

GAPDH

GAPDH+White

White

180

130

100

70

55

40

35

25

15

Supplementary Figure 4V

ZNF419

ZNF419+White

GPX4

GPX4+White

GAPDH

GAPDH+White

Supplementary Figure 5F

ZNF419

ZNF419+White

ZDHHC20

ZDHHC20+White

GPX4

GPX4+White

GAPDH

GAPDH+White

White

70

55

40

35

25

15
